# The causal association between systolic blood pressure and breast cancer: a two sample Mendelian randomization study

**DOI:** 10.1186/s12885-025-15513-x

**Published:** 2026-01-22

**Authors:** Zainab Awada, Nabila Kazmi, Hannah J. Jones, Sarah J. Lewis

**Affiliations:** 1 Department of Population Health Sciences, Bristol Medical School, University of Bristol, Bristol, UK; 2 Medical Research Council Integrative Epidemiology Unit, Population Health Sciences, Bristol Medical School, University of Bristol, Bristol, UK; 3 NIHR Bristol Biomedical Research Centre, University Hospitals Bristol and Weston NHS Foundation Trust, Bristol, UK

**Keywords:** Two-sample Mendelian randomisation, Systolic blood pressure, Breast cancer, Cancer epidemiology

## Abstract

**Background:**

Breast cancer (BC) is the leading cause of cancer incidence worldwide. Observational studies have suggested that hypertension may be a risk factor for BC. We tested whether systolic blood pressure (SBP) may influence BC and BC subtypes using a Mendelian randomisation (MR) approach.

**Methods:**

We used 334 genetic variants associated with SBP as an instrumental variable. Effect estimates were obtained from the UK biobank (*n* = 469,767; SNP-exposure estimates) and the Breast Cancer Association Consortium (BCAC) (122,977 cases and 105,974 controls; SNP-outcome estimates). We assessed BC sub-types including: Triple-negative breast cancer (TNBC), human epidermal growth factor receptor 2 (HER2) positive, Luminal-A, Luminal-B, and Luminal-B HER2 negative. We used inverse variance weighted (IVW) as our primary analysis but conducted a series of sensitivity analyses to test the robustness of our findings. We created a restricted subset of SNPs by excluding SNPs associated with body mass index (BMI) and those with small effect sizes on the exposure, we also conducted multivariable mendelian randomisation analysis to control for adiposity as a potential confounder.

**Results:**

For each 1 mm/Hg increase in SBP the estimated effect was OR 1.00 (0.93, 1.08) for overall BC, analyses of BC sub-types results also did not support a causal effect of SBP. Similarly, results of our sensitivity analyses did not show strong evidence of an association.

**Conclusion:**

The results of our study did not support a causal effect of SBP on overall BC or its sub-types.

**Supplementary Information:**

The online version contains supplementary material available at 10.1186/s12885-025-15513-x.

## Introduction

Breast cancer (BC) has been reported to be the leading cause of global cancer incidence (2.3 million new cases) and the fifth leading cause of cancer mortality in the world, causing 685,000 deaths in 2020. BC is responsible for 1 in 4 cancer cases and 1 in 6 cancer deaths among women [1]. Based on a recent global report on cancer statistics in 2020 [1], BC incidence rates were 88% higher in transitioned than in transitioning countries (55.9 and 29.7 per 100,000, respectively). However, women in transitioning countries had 17% higher mortality rates compared to transitioned countries (15.0 and 12.8 per 100,000, respectively). The higher incidence rates in transitioned countries could be explained by the higher prevalence of reproductive and hormonal risk factors (early menarche, older age at menopause, older age at first birth, fewer children, less breastfeeding, menopause hormonal therapy, oral contraceptives), and other lifestyle risk factors (physical inactivity, alcohol intake, excess body weight) in addition to increased detection of BC cases through higher uptake of mammography screening [2]. Since 2007, there has been a slight increase in incidence rates in the United States and many countries in Europe and Oceania, mainly because of the increased prevalence of risk factors and higher mammography screening rates, but with effective treatment, mortality rates have been dropping since the late 1980s [1].

Hypertension has been inconsistently linked to BC [3]. There is biological plausibility that hypertension could influence BC development through mechanisms involving inflammation and oxidative stress [4]. Hypertension raises inflammation in arteries, and chronic inflammation activates an enzyme called aromatase involved in oestrogen production in adipose tissue in the breast, which creates a perfect microenvironment for breast cancer cells’ growth and invasiveness [4]. It has also been showed that oxidative stress is associated with multiple conditions including cancer and inflammation [4]. A meta-analysis of 30 observational studies showed that hypertensive individuals had a 15% higher risk of BC than non-hypertensive individuals [5]. Higher adult BMI has consistently been shown in observational studies to be a risk factor for postmenopausal breast cancer and protective against premenopausal breast cancer [6]. As blood pressure increases with BMI, it is likely that observational studies of blood pressure are confounded by BMI [7]. The gold standard for assessing the causal effect of hypertension on breast cancer would be through randomised-controlled trials, but these are usually costly and time-consuming [8], and in the case of hypertension as the exposure, it would be unethical to randomize people to have hypertension. For this reason, other methods, such as Mendelian randomization (MR), have been used to assess causality [9]. In MR studies, genetic variants are used as instrumental variables or proxies for exposures of interest and aim to overcome confounding and biases of observational studies [9]. A recent MR study assessing the causal effect of hypertension on total and cite-specific cancer risk (*n* = 757,601 participants of European ancestry) found no evidence of a causal association between hypertension and BC [10]. However, MR studies have found that increased adiposity reduces the risk of breast cancer even in postmenopausal women [11], which may be due to a protective effect of childhood adiposity on BC risk [12]. Given that obesity is known to increase BP, MR analyses which do not exclude BMI-associated instrumental variables are likely to be confounded and BMI could mask an independent effect of BP.

Previous MR studies have also not explored the potential heterogeneity in BC sub-types [10]. Sub-types such as Triple-negative breast cancer (TNBC), Human epidermal growth factor receptor 2 (HER2) positive, Luminal-A, Luminal-B, and Luminal-B HER2 negative have distinct etiologies, molecular characteristics and responses to risk factors [13]; suggesting that hypertension might influence them differently, necessating further analysis [13].

The aim of the present study was to explore the causal effects of systolic blood pressure (SBP) on BC and BC sub-types, including TNBC, HER2 postive, Luminal-A, Luminal-B, and Luminal-B HER2 negative, using Mendelian randomization.

## Methods

This study was conducted in accordance with the Strengthening the Reporting of Observational Studies in Epidemiology using Mendelian Randomization (STROBE-MR) guidelines [14]. All studies included were reviewed and approved by their respective ethics committees and participants included in those studies provided oral or written informed consent.

### Two-sample MR

To investigate the causal effects of SBP on BC, we used a two-sample Mendelian randomization approach [15]. Firstly, genetic instruments as proxies for SBP were selected from summary statistics of a published genome-wide association study (GWAS) of SBP [16]. These were then harmonised with single nucleotide polymorphism (SNP) effect estimates of genetic associations from a published GWAS of overall BC [17] and BC subtypes [18].

### Study populations

#### Blood pressure data

The UK Biobank is a large cohort study that has collected genetic and health information from people living in the UK. From 2006 to 2010, data on half a million participants, aged between 40 and 69 years, have been collected and linked to their health records to help understand causes of disease; participants are still being followed up. Regular assessments take place in 22 centres in England, Wales, and Scotland [19]. A total of 488,377 participants had genotype data available. After removal those with withdrawing consent, we ended up with 488,366. In the UK Biobank cohort, the mean systolic blood pressure (SBP) was 137.7 mm Hg with a standard deviation (SD) of 18.6 mm Hg [16].

A total of 469,767 participants had valid SBP measurements at baseline. Two automated readings of SBP were taken a few moments apart using Omron HEM-7015IT device, an average of the two readings was calculated. For those individuals taking blood pressure medication, this was adjusted for by adding 15 mmHg to the blood pressure measurement [20].

#### Breast cancer consortia data

Summary statistics for genetic associations of BC were obtained from the Breast Cancer Association Consortium (BCAC), and the Discovery, Biology and Risk of Inherited Variants in Breast Cancer Consortium (DRIVE) [17]. There were 122,977 BC cases overall and 105,974 controls of European ancestry (PMID: 29059683), accessed via GWAS Catalog (GCST004988). BC cases in the BCAC consortium were determined by a combination of self-reports, medical/pathology records and cancer registry data [17]. BC cases were stratified into five sub-types: TNBC, HER2 positive, Luminal-A, Luminal-B, and Luminal-B HER2 negative (PMID: 32424353), accessed via the GWAS Catalog (GCST010098) [18]. BC subtypes were determined through data collected from multiple studies within the (BCAC) and the Consortium of Investigators of Modifiers of BRCA1/2 (CIMBA) [18]. The study included 133,384 breast cancer cases and 113,789 controls of European ancestry, as well as 18,908 BRCA1 mutation carriers (9414 with breast cancer); accessed and downloaded in [June 2024]. The determination of breast cancer subtypes was based on diagnoses confirmed by medical records, pathology reports, or cancer registries. Genotyping was performed using the iCOGS array or the OncoArray with imputation (using the version 3 release of the 1000 Genomes Project data set as a reference panel) [18]. Results were combined across the individual studies and consortia using fixed-effect meta-analysis, which resulted in combined dataset including 10,680,257 SNPs.

#### GWAS for positive control outcome

We selected stroke as a positive control outcome to test the validity of our genetic instrument, because we know the risk of stroke should be elevated by greater systolic blood pressure (SBP). The GWAS we used for stroke [21] had a total of 446,696 participants and 40,585 cases (MR-base ID: ebi-a-GCST006906).

#### GWAS for negative control outcome

We used Type 2 Diabetes (T2D) as a negative control outcome because it is known to be strongly linked to BMI, the confounder we are most concerned about, but SBP does not have a direct effect on T2D. The GWAS we used for T2D [22] 70,127 individuals; 12,931 cases; 57,196 controls of European ancestry (MR-base ID: ebi-a-GCST005413).

All GWAS summary statistics used in this study were obtained from publicly available sources that do not require special access or permission for download or use.

### Instrumental variables for exposure

SNPs associated with SBP were extracted from summary data of a GWAS with a sample of 469,767 UK biobank participants (PMID: 29892013), accessed via GWAS Catalog (GCST90029011) [16]. Data were restricted to individuals of European ancestry and downloaded in [June 2024]. We selected 424 SNPs at a genome-wide significance level (*p* < 5 × 10^–8^). Next, these were clumped to linkage disequilibrium (LD) *r*2 < 0.001 (European 1000 Genomes panel), which resulted in 389 SNPs (Suppl. Table 1) being eligible to be used as instrumental variables for the main MR analysis. Of these 389 SNPs, 334 were available in our outcome GWAS [17, 18]. Weights were based on the effect estimates from the SBP GWAS in UKBiobank. The SNP-outcome association was estimated in the BCAC consortium dataset. We only used instruments for SBP, and did not use both diastolic and systolic blood pressure separately because earlier research has shown a strong genetic association (0.93 ± 0.18) and phenotypic correlation (0.74 ± 0.14) between systolic and diastolic blood pressure, suggesting that a significant fraction of genetic variants co-regulate both blood pressure traits [23]. Both SBP and BC effect estimates were derived from large-scale GWAS meta-analyses. Heterogeneity across contributing studies was assessed and reported by the original GWAS authors [16, 17]. This study used a two-sample MR design with non-overlapping samples. The SBP GWAS was based on participants from UK Biobank (*N* = 469,767 individuals of European ancestry), while the breast cancer GWAS included 122,977 cases and 105,974 controls, also of European ancestry. The similarity in ancestry and large sample sizes reduce the risk of bias due to sample heterogeneity.

This MR analysis is based on three key assumptions: (1) Relevance, which assumes the genetic variants are associated with the exposure (SBP), (2) Independence, which states that these genetic variants are not associated with any of the exposure-outcome confounders, and (3) Exclusion restriction, which hold that the genetic instrument affect the outcome (BC) only through their effect on SBP, and not through other biological pathways.

### Statistical analyses

Two-sample MR was used to analyse the causal effects of SBP on the risk of BC, using the “Two-Sample MR” package in RStudio version 4.3.1. SNP–outcome effect estimates were obtained from the BCAC consortium which included study-specific covariates and the top principal components of ancestry to control for population structure. SNP–exposure associations were derived from UK Biobank using BOLT-LMM, which implicitly accounts for population structure and relatedness; sex and age were included as covariates. Our primary analysis method used was the inverse variance-weighted (IVW) MR [24]. For SNPs associated with SBP, Wald ratios were calculated by dividing SNP-outcome association by SNP-exposure association then meta-analysed using the IVW method. Sensitivity analyses included weighted median [25], weighted mode [26], and MR-Egger regression [27], which have different assumptions and can provide valid estimates even when not all instruments are valid. To assess the influence of individual SNPs on results under the IVW analysis, leave-one-out MR analyses were performed [28]. The same analyses were carried out with cancer sub-types as outcomes. Scatter, funnel, and forest plots were also used to visualise all our results.

**Fig. 1:**
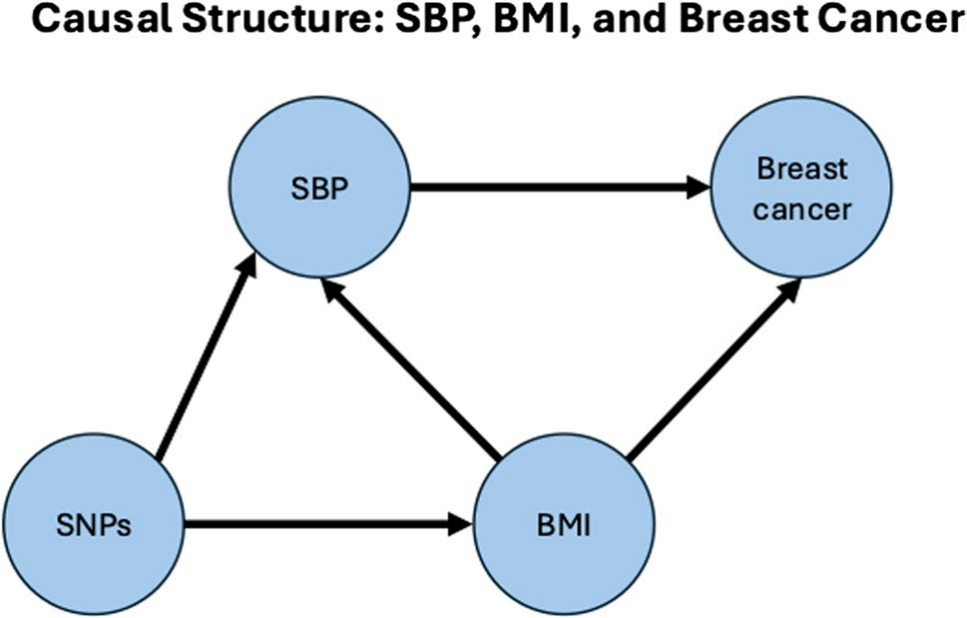
A directed acyclic graph (DAG) to illustrate our causal assumptions, including the potential role of BMI as a confounder and pathway for horizontal pleiotropy

To address the high levels of heterogeneity between SNPs on our outcome, we used MR-Clust [29] to identify any potential clusters in the genetic variants, and Radial MR [30] to detect outliers. Because BMI is associated with both SBP and BC, SNPs for SBP that are also associated with BMI may introduce bias through horizontal pleiotropy (Fig. 1). Therefore, to remove the confounding effect by adiposity, we applied two complementary strategies. First, we created a restricted subset of SNPs in which we only included SNPs with large effect estimates for associations with blood pressure (beta > 0.025 & beta < -0.025) and where we excluded pleiotropic SNPs based on genome-wide significant associations with; body size measures including BMI, sex hormones, lipid traits (e.g. cholesterol) type 2 diabetes and other glucose measures, growth hormones, alcohol, smoking, educational measures, and cancers using data from GWAS Catalog. We further excluded any SNPs associated with BMI at a p-value < 0.05. In our restricted instrument we were left with 54 SNPs, of which 42 were available in our BC outcome GWAS. We repeated our analyses with this restricted set of SNPs and compared our results with those using the full set of SNPs (334 SNPs). Effect estimates were presented per 1 mm/Hg increase in genetically predicted SBP. Second, we additionally performed a multivariable Mendelian randomisation (MVMR) analysis including genetically predicted BMI as a covariate. For the BMI genetic instruments, we used a GWAS conducted in the UK Biobank cohort (MR-base ID: ebi-a-GCST90013974) [31], although previous MR studies of BMI and breast cancer have used a larger meta-analysis of UKBiobank and GIANT consortium [32], we found that the BMI instrument based purely on UKBiobank gave us a greater number of SNPs and an improvement in instrument strength (F-Statistic). We combined genome-wide significant SNPs from both SBP (407 SNPs) and BMI (482 SNPs) datasets, clumped (as above) and then harmonised them with the breast cancer GWAS; this resulted in a final set of 514 SNPs. SNPs were harmonised across datasets and MVMR was performed using the R package “MVMR” [33]. As knowledge of the covariance between the effect of the SNP on each exposure is needed to estimate the MVMR conditional F statistic when using exposure data from overlapping samples [33], covariance matrices were provided using the phenocov_mvmr() function of the MVMR R package. For the SBP and BMI MVMR analysis, a covariance matrix was generated using the phenotypic correlation reported Meena and colleagues [34]. This approach allows appropriate calculation of conditional instrument strength and accounts for the correlation between exposures.

We used publicly available summary-level data from large GWASs for both exposure and outcome. Missing data were handled by the original GWAS analysts, and only SNPs with complete information for both SNP–exposure and SNP–outcome associations were included in the Mendelian Randomization analysis. Even though multiple subgroups were assessed, we did not adjust for multiple testing as these do not represent independent outcomes.

The positive and negative control analyses were performed as described above with SBP as the exposure.

## Results

### Two-sample MR analysis of SBP and overall BC risk

**Fig. 2:**
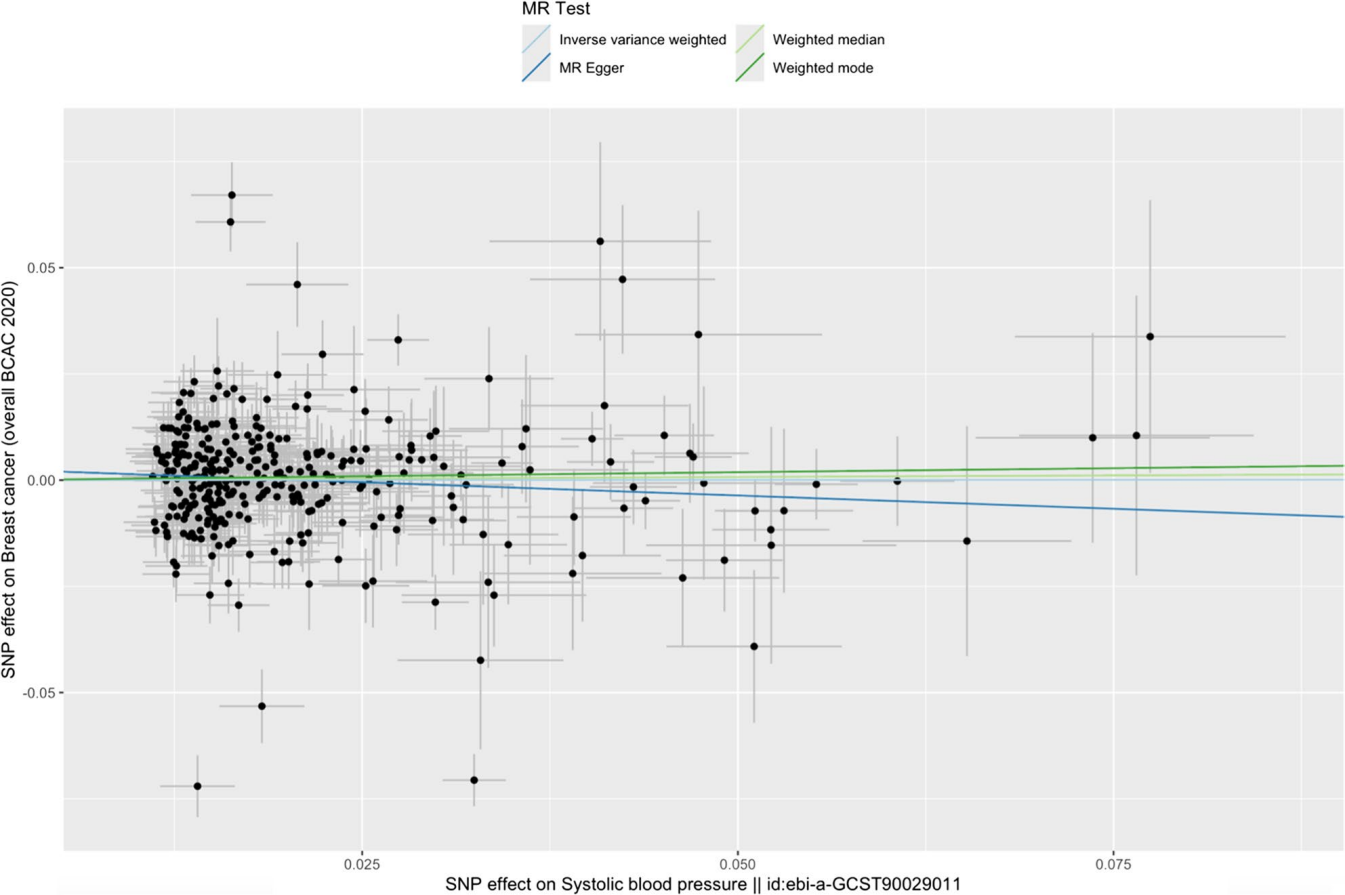
Scatter plot for MR analyses examining the causal effect of full SBP SNPs on BC. Each data point represents the causal effect estimate for an individual SNP

**Fig. 3:**
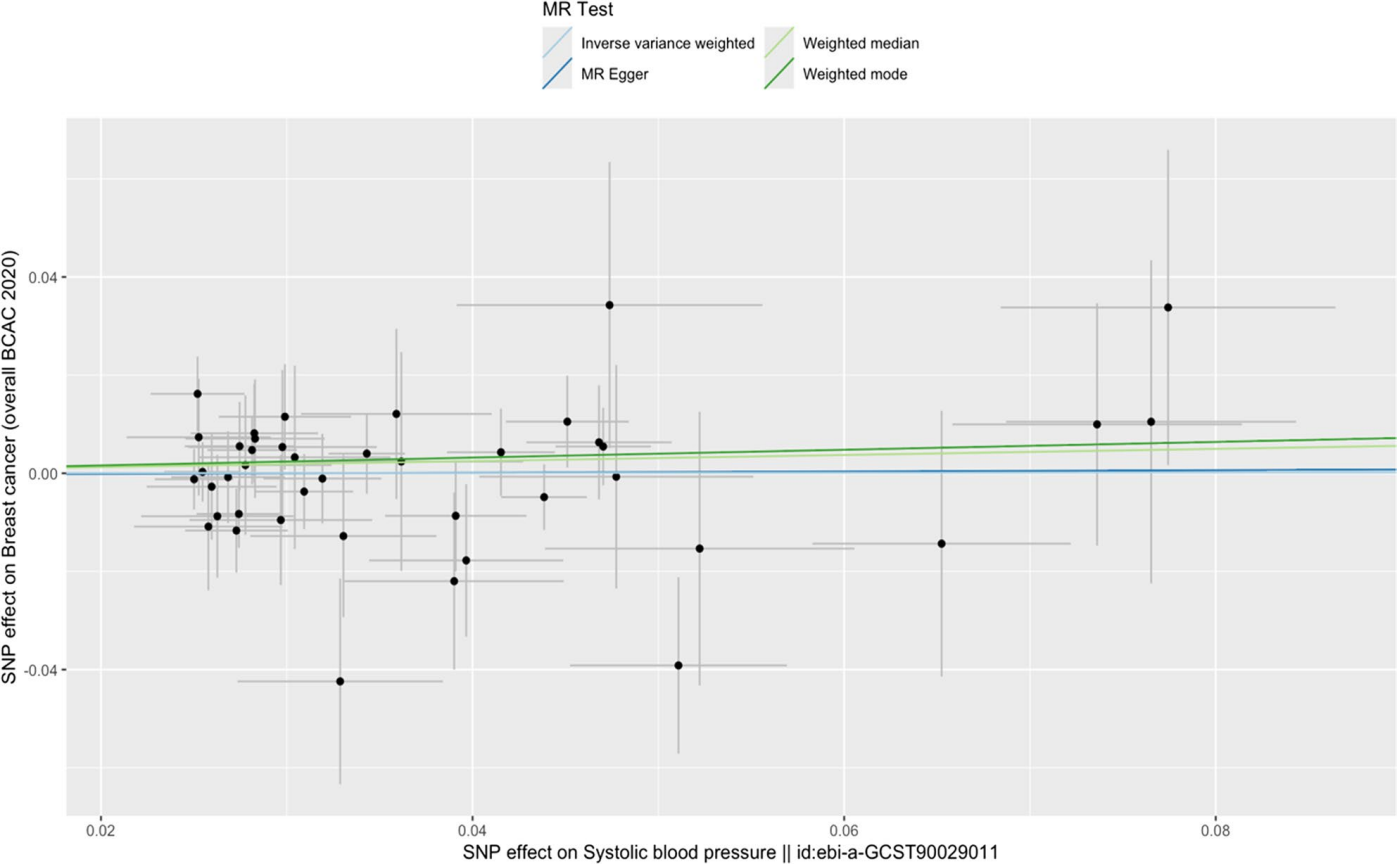
Scatter plot for MR analyses examining the causal effect of restricted SBP SNPs on BC. Each data point represents the causal effect estimate for an individual SNP

The results of the IVW MR using 334 SNPs did not support a causal relationship between SBP and BC risk; OR 1.00 in BC risk per 1 mm/Hg increase in SBP (95% CI 0.93 to 1.08, *p* = 0.97). However, there was strong evidence of heterogeneity between the instrumental variables’ estimates in the IVW MR analysis (Cochranes’s *Q* test *p* = 7.27 × 10^–89^). A series of sensitivity analyses were performed to test the robustness of the IVW estimate and the results of these analyses agreed with the main IVW analysis (Fig. 2 and Table 1). Results using a restricted instrument of 42 SNPs were similar; OR 1.00 in BC risk per 1 mm/Hg increase in SBP (95% CI 0.91 to 1.10, *p* = 0.97) (Table 1, Fig. 3). However, evidence of SNP heterogeneity was weak for this instrument (*p =* 0.80). Effect estimates from sensitivity analyses were all in the same direction except MR Egger; however, confidence intervals for MR Egger overlapped with our IVW analysis, which would suggest this difference in direction is by chance.

**Table 1:** Two-sample MR estimates for the effect of SBP on the risk of overall BC SNPs from GWAS for SBP in UK biobank (*n* = 469,767) per 1 mm/Hg increase in SBP

Method	Full (334 SNPs)				Restricted (54 SNPs)			
	OR	LCI	UCI	*p*-value	OR	LCI	UCI	*p*-value
IVW	1.00	0.93	1.08	0.97	1.00	0.91	1.10	0.97
Weighted median	1.02	0.95	1.09	0.66	1.06	0.93	1.22	0.37
Weighted mode	1.04	0.91	1.18	0.57	1.08	0.89	1.32	0.43
MR-Egger	0.88	0.73	1.07	0.21	1.01	0.71	1.46	0.94
*MR-Egger intercept*	*0.003*			*0.17*	*-0.0004*			*0.95*

**Fig. 4:**
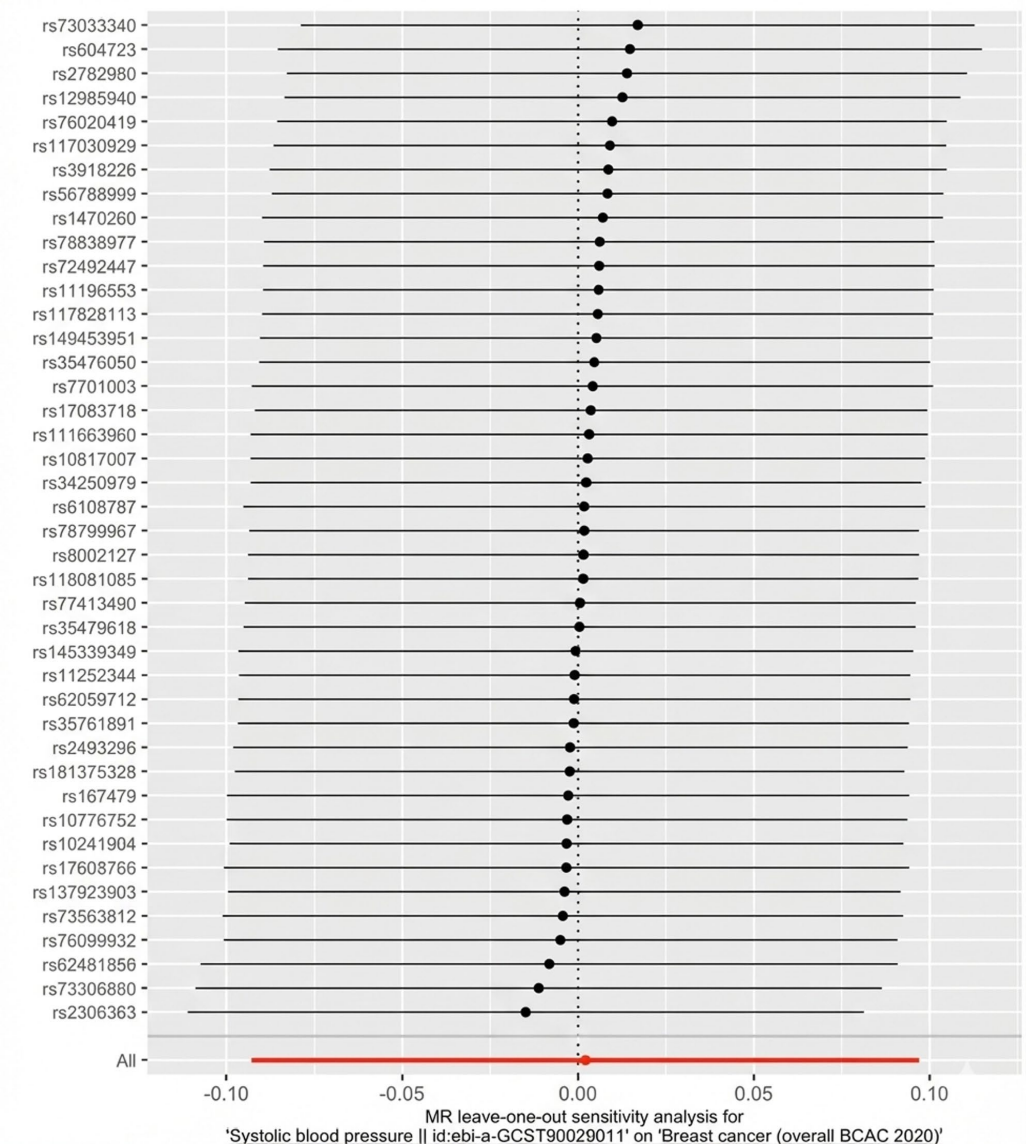
Leave-one-out plot for systolic blood pressure on overall breast cancer

We also performed a leave-one-out analysis for the restricted set of SNPs, and the results suggested no single SNP was disproportionately influencing the results (Fig. 4).

**Fig. 5:**
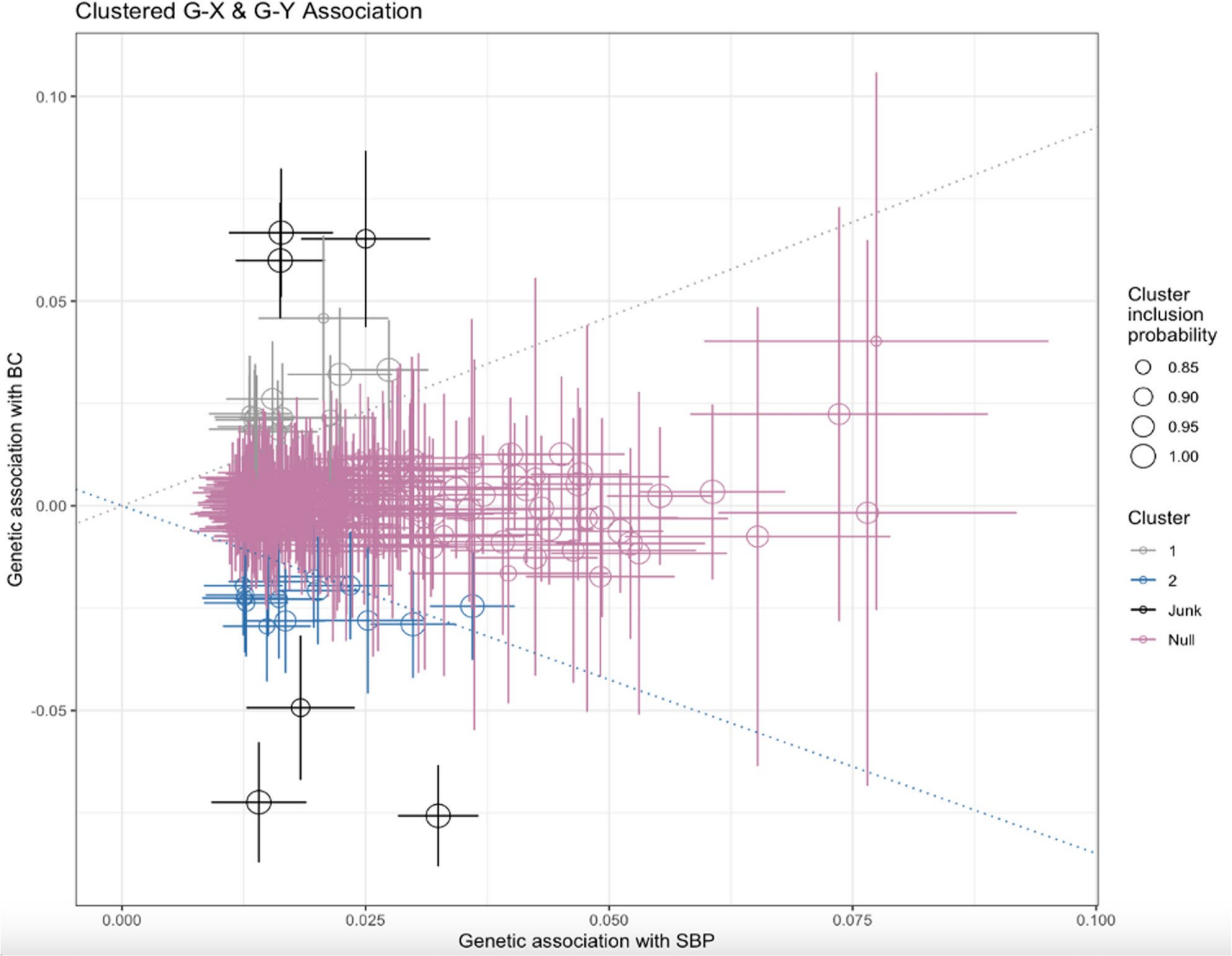
Clustering of genetic variants based on shared associations with blood pressure and breast cancer. Each point represents a SNP’s association with SBP

MRClust analyses [29] identified 3 clusters within our full set of genetic variants (334 SNPs). Clusters were identified if they contained more than 4 SNPs with a cluster inclusion probability of > 0.8. Fig. 5 shows the two effect clusters identified, in addition to the null cluster. One cluster identified contained 12 SNPs which collectively showed a positive association between SBP and BC risk. Using GWAS Catalog, we found that SNPs in this cluster were mainly associated with cardiovascular traits such as systolic and diastolic blood pressure, pulse pressure, and mean arterial pressure, but also showed some evidence of being associated with potential confounders including alcohol drinking, educational attainment, height, and forced expiratory volume (Suppl. Table 3). SNPs (*n* = 14) in the second cluster, which collectively showed a negative association between SBP and BC risk, were also associated with alcohol drinking as well as BMI, type 2 diabetes, lipid traits such as cholesterol, physical activity, smoking, white blood cells traits, cognitive function, sex hormones, etc. SNPs (*n* = 223) in the null cluster did not show an association with BC; they were associated with traits including body size measures (i.e. hip circumference), anxiety, sex hormone measurement, blood lipid traits (i.e. cholesterol), heel bone density, smoking, and white blood cell count (Suppl. Table 3).

**Fig. 6:**
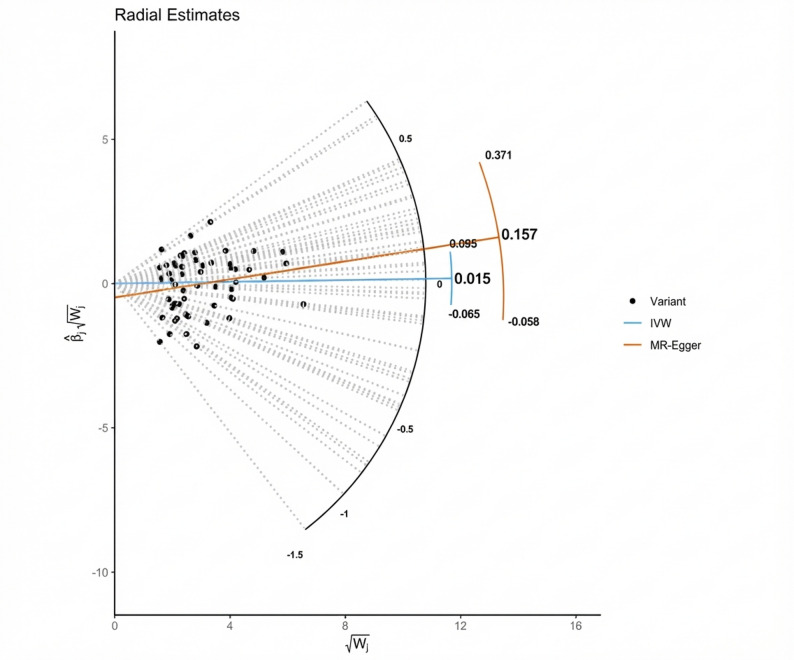
Radial Mendelian randomisation plot identifying outlier SNPs. Each data point represents a genetic variant used in the MR analysis of systolic blood pressure and breast cancer

To further address heterogeneity in the restricted set of SNPs we performed Radial MR [30]; no outliers were detected (Fig. 6).

### Two-sample MR analysis of SBP and BC sub-types

Results of the IVW MR using 334 SNPs did not show strong evidence of a causal effect of SBP on TNBC; OR 1.11 (95% CI 0.97 to 1.26; *p* = 0.14). There was however strong evidence of heterogeneity in the IVW MR (*p* = 1.07 × 10^–16^). The weighted mode and median sensitivity analyses showed some weak evidence of effects on TNBC, although confidence intervals were wide (Table 2, Suppl. Figure 1a). The leave-one-out MR did not suggest a single SNP was driving the results (Supple. Figure 2a). In analyses using the restricted set of 42 SNPs all analyses showed ORs greater than 1 with strongest evidence of effect from the median and mode analyses; OR 1.48 (95% CI 1.04 to 2.11; *p* = 0.03) and 1.58 (95% CI 0.98 to 2.54; *p* = 0.07) respectively, with fairly low p-values (Table 2, Supple. Figure 1b), with no strong evidence of heterogeneity between SNPs (*p =* 0.15).

**Table 2:** Two-sample MR estimates for the effect of SBP on the risk of BC sub-types using SNPs from GWAS for SBP in UK biobank (*n* = 469,767) per 1 mm/Hg increase in SBP

Breast cancer sub-types	Method	Full (334 SNPs)				Restricted (54 SNPs)			
		OR	LCI	UCI	*p-value*	OR	LCI	UCI	*p-value*
TNBC	IVW	1.11	0.97	1.26	0.14	1.09	0.84	1.43	0.50
	Weighted median	1.16	0.99	1.36	0.06	1.48	1.04	2.11	0.03
	Weighted mode	1.51	1.01	2.26	0.05	1.58	0.98	2.54	0.07
	MR-Egger	0.85	0.60	1.22	0.38	1.41	0.51	3.88	0.51
	*MR-Egger intercept*	*0.005*			*0.12*	*-0.009*			*0.62*
HER-2 positive	IVW	0.88	0.75	1.05	0.16	0.95	0.63	1.42	0.79
	Weighted median	0.85	0.66	1.11	0.23	0.86	0.48	1.56	0.24
	Weighted mode	0.73	0.39	1.34	0.31	1.07	0.44	2.60	0.88
	MR-Egger	1.02	0.65	1.62	0.92	2.54	0.55	11.64	0.24
	*MR-Egger intercept*	*-0.003*			*0.50*	*-0.03*			*0.20*
Luminal-A	IVW	1.02	0.93	1.12	0.63	1.05	0.93	1.19	0.44
	Weighted median	1.01	0.93	1.10	0.88	1.04	0.87	1.26	0.64
	Weighted mode	0.97	0.84	1.13	0.71	0.96	0.72	1.28	0.77
	MR-Egger	0.98	0.77	1.24	0.85	1.04	0.65	1.68	0.86
	*MR-Egger intercept*	*0.0009*			*0.69*	*0.0002*			*0.98*
Luminal-B	IVW	0.93	0.81	1.06	0.28	1.11	0.83	1.48	0.48
	Weighted median	0.86	0.71	1.04	0.13	1.11	0.74	1.67	0.62
	Weighted mode	0.71	0.47	1.06	0.09	0.99	0.49	2.01	0.98
	MR-Egger	0.76	0.53	1.09	0.14	2.35	0.79	7.03	0.13
	*MR-Egger intercept*	*0.004*			*0.25*	*-0.03*			*0.17*
Luminal-B HER-2 negative	IVW	0.95	0.84	1.06	0.35	0.86	0.68	1.09	0.20
	Weighted median	0.88	0.74	1.03	0.12	0.85	0.60	1.21	0.36
	Weighted mode	0.82	0.58	1.15	0.24	0.97	0.61	1.55	0.90
	MR-Egger	0.79	0.58	1.08	0.14	0.70	0.28	1.70	0.43
	*MR-Egger intercept*	*0.004*			*0.28*	*0.007*			*0.63*

The IVW MR showed weak evidence of a protective effect of SBP on HER2 positive BC; OR 0.88 (95% CI 0.75 to 1.05; *p* = 0.16); the weighted mode and median sensitivity analyses gave results which were consistent with the IVW, albeit with wide confidence intervals (Table 2, Supple Fig. 1c), and there was no strong evidence of heterogeneity (*p* = 0.29) (Table 2, Supple. Figure 1b). The leave-one-out MR did not provide evidence a single SNP driving the results (Supple. Figure 2b). The restricted sub-set of SNPs showed no strong evidence of a causal effect (Table 2, Supple Fig. 1d).

There was no strong evidence of a causal association between SBP and Luminal-A BC; OR 1.02 (95% CI 0.93 to 1.12; *p* = 0.63). Sensitivity analyses were in agreement with the main IVW (Table 2, Supple Fig. 1e), although there was strong evidence of heterogeneity (*p* = 1.48 × 10^–64^). Results of the restricted SNPs analysis were not different from those of the full SNP analysis (Table 2, Supple Fig. 1f); however, there was no longer evidence of heterogeneity (*p =* 0.73).

No strong evidence was found to support a causal effect of SBP on Luminal-B; OR 0.93 (95% CI 0.81 to 1.06; *p* = 0.28). Sensitivity analyses agreed with the main IVW and showed weak evidence of a protective effect (Table 2, Supple Fig. 1g). As with other sub-types there was strong evidence of heterogeneity between SNP effects (*p* = 6.61 × 10^–7^). Results of the restricted SNPs analysis were not consistent with main set with regards to direction of effect and showed no strong evidence of causal effect (Table 2, Supple Fig. 1h), but again without heterogeneity (*p =* 0.29).

Similarly, there was no strong evidence that SBP had a causal effect on luminal-B HER2 negative BC; IVW OR 0.95 (95% CI 0.84 to 1.06; *p* = 0.35). Sensitivity analyses agreed with the main IVW analysis showing a protective effect of SBP on luminal-B HER2 negative BC (Table 2, Supple Fig. 1i). Again, there was strong evidence of between-SNP-heterogeneity (*p* = 1.36 × 10^–5^). Results of the restricted SNPs analysis were similar to the main analysis (Table 2, Supple Fig. 1j), but without heterogeneity (*p =* 0.82).

### Multivariable MR analysis

Using MVMR with the 514-SNP union set, and adjusting for BMI, we found no strong evidence of a direct effect of SBP on BC (OR per 1 mmHg = 0.99, 95% CI 0.93–1.05, *p* = 0.69). Similarly, there was no strong evidence of an independent effect of BMI on BC (OR per 1 SD = 0.95, 95% CI 0.85–1.07, *p* = 0.35). The conditional F-statistics (SBP F = 44.05; BMI F = 15.98) indicated adequate instrument strength for both exposures. We also performed a univariate analysis of BMI and breast cancer using the same instrument and found weak evidence of a protective effect (OR = 0.96, 95% CI 0.89–1.03, *p* = 0.32).

### Positive control analysis

The results of our IVW positive control outcome analysis using 319 SNPs suggested that a 1 mm/Hg increase in SBP increases the risk of stroke; OR 1.66 (95% CI 1.55 to 1.78, *p* = 6.57 × 10^–46^), with strong evidence of heterogeneity (*p* = 2.84 × 10^–14^). For the restricted SNP instrument, the IVW results using 36 SNPs suggested that a 1 mm/Hg increase in SBP increases the risk of stroke; OR 1.96 (95% CI 1.60 to 2.40, *p* = 5.44 × 10^–11^); although the heterogeneity was reduced somewhat, we still found strong evidence of between SNP heterogeneity (*p* = 6.63 × 10^–6^).

### Negative control analysis

We tested the associations between the full (390 SNPs) and restricted (42 SNPs) sets of SBP SNPs and T2D in negative control analyses. Using the T2D dataset for the outcome [22], the IVW analysis suggested that there was weak evidence of an association; OR 0.94 (95% CI 0.83 to 1.07, *p* = 0.36) when using the full set of SNPs. Similarly, the IVW using the restricted set of SNPs did not show evidence of an association; OR 1.01 (95% CI 0.83 to 1.37, *p* = 0.62) (Suppl. Table 4).

## Discussion

To assess the causal effect of SBP on the risk of BC, we performed a 2-sample Mendelian randomisation analysis. We used genetic variants identified by a GWAS of blood pressure traits in the UK biobank (*n* = 469,767). No strong evidence was found to support the hypothesis of SBP having a causal effect on BC.

Our results are in line with the findings of a meta-analysis of observational studies [3] and a previous Mendelian randomisation study assessing the causal effects of systolic and diastolic blood pressure traits on 17 cancers including BC, both of which found no evidence of an effect [10]. With regard to BC sub-types, our findings did not provide strong evidence of a causal association between any sub-type and SBP, which is in agreement with another prospective cohort study [35], although we did find some evidence of a positive effect of SBP on TNBC across sensitivity analyses. These results should be interpreted with caution given the reduced number of cases available for the TNBC analysis which limited statistical power as shown by the wide confidence intervals.

In contrast, a systematic review and meta-analysis of 30 observational studies by Han et al. [5] found that hypertensive women had a higher risk of BC compared to non-hypertensive women. This discrepancy between the meta-analysis and our results could be explained by reverse causality and/ or residual confounding in observational studies, issues which are less likely to influence MR studies because genetic variants are randomly assorted at conception [9]. Even in the observational studies when a subgroup analysis was performed, researchers found that this association was only apparent in postmenopausal women. Since BMI has been shown to increase the risk of breast cancer among postmenopausal women and decrease the risk of breast cancer among premenopausal women, the effect seen in the meta-analysis of observational studies could have arisen due to confounding by BMI. Previous MR studies have reported an inverse association between adult adiposity and premenopausal breast cancer [11], and between childhood adiposity and all breast cancers [12]. Because a high BMI is strongly associated with an increased BP, MR studies which failed to investigate the effect of their SBP instrument on BMI could fail to detect an independent effect of SBP on BC due to confounding by BMI.

Our initial analyses using 334 SNPs from the UK biobank found strong evidence of heterogeneity between SNPs. We investigated this heterogeneity using additional analyses such as MR-Clust and Radial MR. We also conducted analyses using a reduced SNP instrument, keeping just the SNPs having a substantial effect on SBP and removing any SNPs which had been found to be associated with risk factors for BC in GWAS, or were associated with BMI. When we used our reduced SNP instrument, there was no longer heterogeneity between SNPs for the effect on BC, but the results were largely unchanged.

The main analysis method we used was IVW MR, which assumes that all instrumental variables (genetic variants) are valid; otherwise, results would be biased. To overcome this, we performed a series of sensitivity analyses (MR-Egger, MR PRESSO, weighted-median, and weighted-mode) which each has different assumptions. None of the sensitivity analyses’ results provided very strong, consistent evidence of an association between SBP and overall BC risk.

In MRClust analysis, SNPs in a cluster share similar patterns in their genetic associations with both SBP and BC. Three clusters of SNPs were identified each with different directions of effect, which could suggest multiple biological pathways linking SBP to BC. Our analysis suggests that different confounding factors or biological pathways may be having opposing effects on BC. Some of the main traits associated with SBP SNPs were BMI, lipid traits, cancers, body size measures, and hormonal factors. Since these factors might act as potential confounders, SNPs associated with them were excluded from the restricted subset of SNPs in an attempt to reduce any potential confounding effect on the association between SBP and BC.

To further assess confounding by BMI, we performed an MVMR analysis. Previous MR studies have reported an inverse association between BMI and breast cancer risk [36], Similarly, our MVMR analysis produced inverse associations of similar magnitudes. Our UKB-based MVMR found weak evidence of a small BMI effect (OR = 0.95, 95% CI 0.85–1.07, *p* = 0.35), and the univariate analysis using the same instrument gave a similar result (OR = 0.96, 95% CI 0.89–1.03, *p* = 0.32) consistent with a previous MR study that assessed BMI and BC using a different set of BMI instruments from a GWAS by Yengo L et al. [36], (OR = 0.96, 95% CI 0.91, 1.00). We explored using the same instrument in our study but the F-Stat for the instrument by Yengo et al., was 7.1 for the MVMR, which meant that the analysis would suffer from weak instrument bias.

The relationship between SBP and BC is biologically plausible through pathways such as inflammation and oxidative stress, but remains uncertain [4]. Observational studies reported positive associations between SBP and BC, but these may be confounded as discussed previously. In this two-sample MR study, we found no association between SBP and BC or BC subtypes. Our findings suggest that associations found in observational studies may reflect confounding or reverse causality rather than a direct causal relationship.

### Limitations

Although MR analysis overcomes some of the biases of observational studies, it still has its own weaknesses. MR assumes a linear relationship between the continuous exposure and the outcome, so if the association between the continuous measure of SBP and BC in our study was non-linear, it would not be detected.

We took many steps to try to rule out any effect of masking by BMI and other potential confounders in our analyses, i.e. by using a reduced set of SNPs which have large effects on SBP and are not associated with potential confounders and performing an MVMR analysis, despite this it is not possible to completely rule out confounding.

Genetic proxies for SBP used in our analyses were not sex-specific; therefore, a female-specific GWAS is needed to identify female-specific SNPs that proxy SBP. However, we evaluated the consistency of SNP effect estimates between our combined-sex SBP GWAS and a male-specific SBP GWAS [23]. The strong positive correlation observed (*r* = 0.80, *p* < 7.6 × 10⁻¹²) suggests that the genetic instruments derived from the combined-sex GWAS are robust and broadly representative, supporting their use in this sex-specific (female) outcome analysis. Furthermore, given the geographical and ethnic differences in the prevalence of BC, and since our study was limited to participants of European ancestry, more MR studies would enrich the current body of evidence regarding the association between blood pressure and BC in different populations and ethnic groups.

In conclusion, our MR results suggested that SBP does not have a causal effect on BC overall with no strong evidence for any of the BC sub-types, although suggestive evidence of a positive effect on TNBC. Our results were consistent across sensitivity analyses. Future research should focus on studying this association in pre-menopausal and postmenopausal women and for populations of different ancestries other than Europeans before an effect of blood pressure on BC risk can be completely ruled out. 

## Supplementary Information

Supplementary Material 1.

Supplementary Material 2.

## Data Availability

This work has been done using the UK Biobank Resource. The UK Biobank is an open-access resource and researchers can use the UK Biobank dataset by applying and registering at [http://ukbiobank.ac.uk/register-apply/](http:/ukbiobank.ac.uk/register-apply) .
