# Supplementary material for: The causal association between systolic blood pressure and breast cancer: a two sample Mendelian randomization study
Source: BMC Cancer. 2026 Jan 22;26:264. doi: 10.1186/s12885-025-15513-x (PMC12914972; doi:10.1186/s12885-025-15513-x)
Supplement: Supplementary file 1 — Supplementary Material 1. [file 12885_2025_15513_MOESM1_ESM.docx]

**STROBE-MR checklist of recommended items to address in reports of Mendelian randomization studies**^1^ ^2^

| **Item No.** | **Section** | **Checklist item** | **Page No.** | **Relevant text from manuscript** |
| --- | --- | --- | --- | --- |
| 1 | **TITLE and ABSTRACT** | Indicate Mendelian randomization (MR) as the study’s design in the title and/or the abstract if that is a main purpose of the study | 1 | Title: “The causal association between systolic blood pressure and breast cancer: A two sample Mendelian Randomisation study”  Abstract: “We tested whether systolic blood pressure (SBP) may influence BC and BC subtypes using a Mendelian randomisation (MR) approach. We used 334 genetic variants associated with SBP as an instrumental variable.” |
|  | **INTRODUCTION** |  |  |  |
| 2 | **Background** | Explain the scientific background and rationale for the reported study. What is the exposure? Is a potential causal relationship between exposure and outcome plausible? Justify why MR is a helpful method to address the study question | 3 | “A meta-analysis of 30 observational studies showed that hypertensive individuals had a 15% higher risk of BC than non-hypertensive individuals.” “The gold standard for assessing the causal effect of hypertension on breast cancer would be through randomised-controlled trials, but these are usually costly and time-consuming, and in the case of hypertension as the exposure, it would be unethical to randomize people to have hypertension.” “In MR studies, genetic variants are used as instrumental variables or proxies for exposures of interest and aim to overcome confounding and biases of observational studies” |
| 3 | **Objectives** | State specific objectives clearly, including pre-specified causal hypotheses (if any). State that MR is a method that, under specific assumptions, intends to estimate causal effects | 4 | “The aim of the present study was to explore the causal effects of systolic blood pressure (SBP) on BC and BC sub-types, including TNBC, HER2, Luminal-A, Luminal-B, and Luminal-B HER2 negative, using Mendelian randomisation.” |
|  | **METHODS** |  |  |  |
| 4 | **Study design and data sources** | Present key elements of the study design early in the article. Consider including a table listing sources of data for all phases of the study. For each data source contributing to the analysis, describe the following: | 4-6 | These are described in the Methods.   - SBP GWAS ID (GCST90029011) - BC GWAS ID (GCST004988)/ subtypes (GCST010098) |
|  | a) | Setting: Describe the study design and the underlying population, if possible. Describe the setting, locations, and relevant dates, including periods of recruitment, exposure, follow-up, and data collection, when available. | 5-6 | All study populations used in the analysis are described in the Methods. |
|  | b) | Participants: Give the eligibility criteria, and the sources and methods of selection of participants. Report the sample size, and whether any power or sample size calculations were carried out prior to the main analysis | 5 | Sample sizes of the studies used are described in the text. |
|  | c) | Describe measurement, quality control and selection of genetic variants | 7 | “SNPs associated with SBP were extracted from summary data of a GWAS with a sample of 469,767 UK biobank participants (PMID: 29892013), accessed via GWAS Catalog (GCST90029018). Data were restricted to individuals of European ancestry and downloaded in [June 2024]. We selected 424 SNPs at a genome-wide significance level (p < 5 × 10–8). Next, these were clumped to linkage disequilibrium (LD) r2 < 0.001 (European 1000 Genomes panel), which resulted in 389 SNPs (Suppl. table 1) being eligible to be used as instrumental variables for the main MR analysis. Of these 389 SNPs, 334 were available in our outcome GWAS.” |
|  | d) | For each exposure, outcome, and other relevant variables, describe methods of assessment and diagnostic criteria for diseases | 5-6 | “A total of 469,767 participants had valid SBP measurements at baseline. Two automated readings of SBP were taken a few moments apart using Omron HEM-7015IT device, an average of the two readings was calculated. For those individuals taking blood pressure medication this was adjusted for by adding 15 mmHg to the blood pressure measurement.” “Summary statistics for genetic associations of BC were obtained from the Breast Cancer Association Consortium (BCAC), and the Discovery, Biology and Risk of Inherited Variants in Breast Cancer Consortium (DRIVE).” “BC cases in the BCAC consortium were determined by a combination of self-reports, medical/pathology records and cancer registry data.” “The determination of breast cancer cases was based on diagnoses confirmed by medical records, pathology reports, or cancer registries.” |
|  | e) | Provide details of ethics committee approval and participant informed consent, if relevant | 4 | “All studies included were reviewed and approved by their respective ethics committees and participants included in those studies provided oral or written informed consent.” |
| 5 | **Assumptions** | Explicitly state the three core IV assumptions for the main analysis (relevance, independence and exclusion restriction) as well assumptions for any additional or sensitivity analysis | 8 | “This MR analysis is based on three key assumptions: 1. Relevance, which assumes the genetic variants are associated with the exposure (SBP), 2. Independence, which states that these genetic variants are not associated with any of the exposure-outcome confounders, and 3. Exclusion restriction, which hold that the genetic instrument affect the outcome (BC) only through their effect on SBP, and not through other biological pathways.” |
| 6 | **Statistical methods: main analysis** | Describe statistical methods and statistics used | 8 | “Our primary analysis method used was the inverse variance-weighted (IVW) MR. Sensitivity analyses included weighted median, weighted mode, and MR-Egger regression” “To assess the influence of individual SNPs on results under the IVW analysis, leave-one-out MR analyses were performed” |
|  | a) | Describe how quantitative variables were handled in the analyses (i.e., scale, units, model) | 9 | “Effect estimates were presented per 1 mm/Hg increase in genetically predicted SBP.” |
|  | b) | Describe how genetic variants were handled in the analyses and, if applicable, how their weights were selected | 7 | “Weights were based on the effect estimates from the SBP GWAS in UKBiobank.” |
|  | c) | Describe the MR estimator (e.g. two-stage least squares, Wald ratio) and related statistics. Detail the included covariates and, in case of two-sample MR, whether the same covariate set was used for adjustment in the two samples | 8 | “SNP–outcome effect estimates were obtained from the BCAC consortium which study-specific covariates and the top principal components of ancestry to control for population structure. SNP–exposure associations were derived from UK Biobank using BOLT-LMM, which implicitly accounts for population structure and relatedness; sex and age were included as covariates.” “For SNPs associated with SBP, Wald ratios were calculated by dividing SNP-outcome association by SNP-exposure association them meta-analysed using the IVW method.” |
|  | d) | Explain how missing data were addressed | 10 | “We used publicly available summary-level data from large GWASs for both exposure and outcome. Missing data were handled by the original GWAS analysts, and only SNPs with complete information for both SNP–exposure and SNP–outcome associations were included in the Mendelian Randomization analysis.” |
|  | e) | If applicable, indicate how multiple testing was addressed | 10 | “Even though multiple subgroups were assessed, we did not adjust for multiple testing as these do not represent independent outcomes.” |
| 7 | **Assessment of assumptions** | Describe any methods or prior knowledge used to assess the assumptions or justify their validity | 6,9 | “We selected stroke as a positive control outcome” “We used BMI as a negative control outcome” page 6  “We selected 424 SNPs at a genome-wide significance level (p < 5 × 10–8). Next, these were clumped to linkage disequilibrium (LD) r2 < 0.001 (European 1000 Genomes panel), which resulted in 389 SNPs (Suppl. table 1) being eligible to be used as instrumental variables for the main MR analysis.” “Sensitivity analyses included weighted median, weighted mode, and MR-Egger regression, which have different assumptions and can provide valid estimates even when not all instruments are valid.” Pages 7-8  “to remove the confounding effect by adiposity, we applied two complementary strategies. First, we created a restricted subset of SNPs in which we only included SNPs..” “Second, we additionally performed a multivariable Mendelian randomisation (MVMR) analysis including genetically predicted BMI as a covariate.” Pages 8-9 |
| 8 | **Sensitivity analyses and additional analyses** | Describe any sensitivity analyses or additional analyses performed (e.g. comparison of effect estimates from different approaches, independent replication, bias analytic techniques, validation of instruments, simulations) | 8 | “Sensitivity analyses included weighted median, weighted mode, and MR-Egger regression, which have different assumptions and can provide valid estimates even when not all instruments are valid. To assess the influence of individual SNPs on results under the IVW analysis, leave-one-out MR analyses were performed{Burgess, 2017 #60}.”  “To address the high levels of heterogeneity between SNPs on our outcome, we used MR-Clust to identify any potential clusters in the genetic variants, and Radial MR to detect outliers.” |
| 9 | **Software and pre-registration** |  |  |  |
|  | a) | Name statistical software and package(s), including version and settings used | 8 | “Two-sample MR was used to analyse the causal effects of SBP on the risk of BC, using the “Two-Sample MR” package in RStudio version 4.3.1.” “To address the high levels of heterogeneity between SNPs on our outcome, we used MR-Clust to identify any potential clusters in the genetic variants, and Radial MR to detect outliers.” |
|  | b) | State whether the study protocol and details were pre-registered (as well as when and where) |  | Not applicable |
|  | **RESULTS** |  |  |  |
| 10 | **Descriptive data** |  |  |  |
|  | a) | Report the numbers of individuals at each stage of included studies and reasons for exclusion. Consider use of a flow diagram | 5-6 | “A total of 488,377 participants had genotype data available. After removal those with withdrawing consent, we ended up with 488,366. A total of 469,767 participants had SBP measured at baseline.”  “There were 122,977 BC cases overall and 105,974 controls”  “The study included 133,384 breast cancer cases and 113,789 controls of European ancestry, as well as 18,908 BRCA1 mutation carriers (9,414 with breast cancer)” |
|  | b) | Report summary statistics for phenotypic exposure(s), outcome(s), and other relevant variables (e.g. means, SDs, proportions) | 5 | “In the UK Biobank cohort, the mean systolic blood pressure (SBP) was 137.7 mm Hg with a standard deviation (SD) of 18.6 mm Hg.”  See supplementary table 1 |
|  | c) | If the data sources include meta-analyses of previous studies, provide the assessments of heterogeneity across these studies | 7 | “Both SBP and BC effect estimates were derived from large-scale GWAS meta-analyses. Heterogeneity across contributing studies was assessed and reported by the original GWAS authors.” |
|  | d) | For two-sample MR:  i.  Provide justification of the similarity of the genetic variant-exposure associations between the exposure and outcome samples  ii.  Provide information on the number of individuals who overlap between the exposure and outcome studies | 7 | “The SBP GWAS was based on participants from UK Biobank (N = 469,767 individuals of European ancestry), while the breast cancer GWAS included 122,977 cases and 105,974 controls, also of European ancestry. Although both GWAS meta-analyses included European-ancestry individuals, there was no known participant overlap between the exposure and outcome datasets. The similarity in ancestry and large sample sizes reduce the risk of bias due to sample heterogeneity.” |
| 11 | **Main results** |  |  |  |
|  | a) | Report the associations between genetic variant and exposure, and between genetic variant and outcome, preferably on an interpretable scale |  | See supplementary table 1 for instrument-exposure associations.  Due to the large number of individual SNPs in the instruments used, the SNP-outcome associations are represented on scatter plots presented in Figure 1 for overall BC, and supplementary figures for BC subtypes. |
|  | b) | Report MR estimates of the relationship between exposure and outcome, and the measures of uncertainty from the MR analysis, on an interpretable scale, such as odds ratio or relative risk per SD difference | 10 | “The results of the IVW MR using 334 SNPs did not support a causal relationship between SBP and BC risk; OR 1.00 in BC risk per 1 mm/Hg increase in SBP (95% CI 0.93 to 1.08, p = 0.97).” “A series of sensitivity analyses were performed to test the robustness of the IVW estimate and the results of these analyses agreed with the main IVW analysis (Figure 2 and Table 1). Results using a restricted instrument of 42 SNPs were similar; OR 1.00 in BC risk per 1 mm/Hg increase in SBP (95% CI 0.91 to 1.10, p = 0.97) (Table 1, figure 3).” |
|  | c) | If relevant, consider translating estimates of relative risk into absolute risk for a meaningful time period |  | Not applicable |
|  | d) | Consider plots to visualize results (e.g. forest plot, scatterplot of associations between genetic variants and outcome versus between genetic variants and exposure) |  | See figures 2,3 uploaded separately |
| 12 | **Assessment of assumptions** |  |  |  |
|  | a) | Report the assessment of the validity of the assumptions | 8 | “Sensitivity analyses included weighted median, weighted mode, and MR-Egger regression, which have different assumptions and can provide valid estimates even when not all instruments are valid. To assess the influence of individual SNPs on results under the IVW analysis, leave-one-out MR analyses were performed"  See tables 1,2 for sensitivity analyses results  See supplementary table 1 for SNP-exposure associations |
|  | b) | Report any additional statistics (e.g., assessments of heterogeneity across genetic variants, such as *I^2^*, Q statistic or E-value) | 10 | “However, there was strong evidence of heterogeneity between the instrumental variables’ estimates in the IVW MR analysis (Cochranes’s Q test p = 7.27 × 10^–89^).’ |
| 13 | **Sensitivity analyses and additional analyses** |  |  |  |
|  | a) | Report any sensitivity analyses to assess the robustness of the main results to violations of the assumptions | 8 | “Sensitivity analyses included weighted median, weighted mode, and MR-Egger regression, which have different assumptions and can provide valid estimates even when not all instruments are valid. To assess the influence of individual SNPs on results under the IVW analysis, leave-one-out MR analyses were performed{Burgess, 2017 #60}.”  “To address the high levels of heterogeneity between SNPs on our outcome, we used MR-Clust to identify any potential clusters in the genetic variants, and Radial MR to detect outliers.” |
|  | b) | Report results from other sensitivity analyses or additional analyses |  | See tables 1,2 |
|  | c) | Report any assessment of direction of causal relationship (e.g., bidirectional MR) |  | Not applicable |
|  | d) | When relevant, report and compare with estimates from non-MR analyses |  | Not applicable |
|  | e) | Consider additional plots to visualize results (e.g., leave-one-out analyses) |  | See supplementary materials document |
|  | **DISCUSSION** |  |  |  |
| 14 | **Key results** | Summarize key results with reference to study objectives | 14 | “No strong evidence was found to support the hypothesis of SBP having a causal effect on BC.” |
| 15 | **Limitations** | Discuss limitations of the study, taking into account the validity of the IV assumptions, other sources of potential bias, and imprecision. Discuss both direction and magnitude of any potential bias and any efforts to address them | 17 | “MR assumes a linear relationship between the continuous exposure and the outcome, so if the association between the continuous measure of SBP and BC in our study was non-linear, it would not be detected. We took many steps to try to rule out any effect of masking by BMI and other potential confounders in our analyses, i.e. by using a reduced set of SNPs which have large effects on SBP and are not associated with potential confounders and performing an MVMR analysis, despite this it is not possible to completely rule out confounding. Genetic proxies for SBP used in our analyses were not sex specific.” |
| 16 | **Interpretation** |  |  |  |
|  | a) | Meaning: Give a cautious overall interpretation of results in the context of their limitations and in comparison with other studies | 18 | “In conclusion, our MR results suggested that SBP does not have a causal effect on BC overall with no strong evidence for any of the BC sub-types, although suggestive evidence of a positive effect on TNBC. Our results were consistent across sensitivity analyses. Future research should focus on studying this association in pre-menopausal and postmenopausal women and for populations of different ancestries other than Europeans before an effect of blood pressure on BC risk can be completely ruled out.” |
|  | b) | Mechanism: Discuss underlying biological mechanisms that could drive a potential causal relationship between the investigated exposure and the outcome, and whether the gene-environment equivalence assumption is reasonable. Use causal language carefully, clarifying that IV estimates may provide causal effects only under certain assumptions | 17 | “The relationship between SBP and BC is biologically plausible through pathways such as inflammation and oxidative stress but remains uncertain. Observational studies reported positive associations between SBP and BC, but these may be confounded as discussed previously. In this two-sample MR study, we found no association between SBP and BC or BC subtypes. Although we selected genetic instruments strongly associated with SBP and performed multiple sensitivity analyses to evaluate MR assumptions, pleiotropy and other violations cannot be fully ruled out. Our findings suggest that associations found in observational studies may reflect confounding or reverse causality rather than a direct causal relationship.” |
|  | c) | Clinical relevance: Discuss whether the results have clinical or public policy relevance, and to what extent they inform effect sizes of possible interventions | 18 | “Future research should focus on studying this association in pre-menopausal and postmenopausal women and for populations of different ancestries other than Europeans before an effect of blood pressure on BC risk can be completely ruled out.” |
| 17 | **Generalizability** | Discuss the generalizability of the study results (a) to other populations, (b) across other exposure periods/timings, and (c) across other levels of exposure | 17-18 | “Genetic proxies for SBP used in our analyses were not sex-specific; therefore, a female-specific GWAS is needed to identify female-specific SNPs that proxy SBP. Furthermore, given the geographical and ethnic differences in the prevalence of BC, and since our study was limited to participants of European ancestry, more MR studies would enrich the current body of evidence regarding the association between blood pressure and BC in different populations and ethnic groups.” |
|  | **OTHER INFORMATION** |  |  |  |
| 18 | **Funding** | Describe sources of funding and the role of funders in the present study and, if applicable, sources of funding for the databases and original study or studies on which the present study is based | 19 | “This work was supported by Cancer Research UK [grant number C18281/A29019].” |
| 19 | **Data and data sharing** | Provide the data used to perform all analyses or report where and how the data can be accessed, and reference these sources in the article. Provide the statistical code needed to reproduce the results in the article, or report whether the code is publicly accessible and if so, where | 18 | “This work has been done using the UK Biobank Resource. The UK Biobank is an open-access resource and researchers can use the UK Biobank dataset by applying and registering at http://ukbiobank.ac.uk/register-apply/.” |
| 20 | **Conflicts of Interest** | All authors should declare all potential conflicts of interest | 18 | “The authors declare no competing interests.” |

This checklist is copyrighted by the Equator Network under the Creative Commons Attribution 3.0 Unported (CC BY 3.0) license.

1. Skrivankova VW, Richmond RC, Woolf BAR, Yarmolinsky J, Davies NM, Swanson SA, et al. Strengthening the Reporting of Observational Studies in Epidemiology using Mendelian Randomization (STROBE-MR) Statement. JAMA. 2021;under review.

2. Skrivankova VW, Richmond RC, Woolf BAR, Davies NM, Swanson SA, VanderWeele TJ, et al. Strengthening the Reporting of Observational Studies in Epidemiology using Mendelian Randomisation (STROBE-MR): Explanation and Elaboration. BMJ. 2021;375:n2233.
