# Supplementary material for: The causal association between systolic blood pressure and breast cancer: a two sample Mendelian randomization study
Source: BMC Cancer. 2026 Jan 22;26:264. doi: 10.1186/s12885-025-15513-x (PMC12914972; doi:10.1186/s12885-025-15513-x)

**SUPPLEMENTARY MATERIALS**

**Supplementary tables**

**Supplementary table 1. genetic variants used as instrumental variables in the main analysis.**

*Chr: chromosome, EAF: effect allele frequency, SE: standard error

| SNP | Effect allele | Other allele | chr | EAF | Beta | SE | p-value | F-statistics |
| --- | --- | --- | --- | --- | --- | --- | --- | --- |
| rs55857306 | A | G | 1 | 0.16 | -0.06 | 0.003 | 4.50E-87 | 399.14 |
| rs181375328 | A | G | 1 | 0.02 | -0.05 | 0.008 | 4.30E-09 | 33.19 |
| rs4293010 | T | G | 1 | 0.93 | 0.02 | 0.004 | 4.00E-08 | 30.62 |
| rs182050989 | T | C | 1 | 0.03 | 0.04 | 0.006 | 1.80E-11 | 47.19 |
| rs35479618 | A | G | 1 | 0.02 | 0.08 | 0.008 | 8.80E-24 | 96.17 |
| rs12731646 | T | C | 1 | 0.41 | -0.01 | 0.002 | 6.60E-11 | 41.73 |
| rs4382763 | A | C | 1 | 0.21 | 0.01 | 0.002 | 2.70E-08 | 30.20 |
| rs10429910 | C | A | 1 | 0.14 | 0.02 | 0.003 | 1.20E-10 | 39.17 |
| rs2076328 | T | G | 1 | 0.50 | -0.02 | 0.002 | 6.00E-24 | 99.07 |
| rs2493296 | T | C | 1 | 0.14 | 0.03 | 0.003 | 2.80E-21 | 86.34 |
| rs4320727 | A | G | 1 | 0.63 | 0.02 | 0.002 | 7.50E-14 | 53.49 |
| rs61780439 | A | G | 1 | 0.22 | -0.01 | 0.002 | 4.80E-08 | 27.32 |
| rs10890181 | T | C | 1 | 0.49 | -0.02 | 0.002 | 1.70E-15 | 63.62 |
| rs2782652 | C | T | 1 | 0.45 | 0.02 | 0.002 | 2.80E-19 | 78.70 |
| rs6683704 | C | T | 1 | 0.65 | -0.01 | 0.002 | 1.90E-10 | 38.75 |
| rs55869368 | T | C | 1 | 0.09 | -0.02 | 0.004 | 5.90E-10 | 36.86 |
| rs3766612 | A | C | 1 | 0.20 | -0.02 | 0.003 | 4.10E-09 | 36.57 |
| rs11117781 | G | A | 1 | 0.66 | -0.01 | 0.002 | 3.90E-09 | 31.69 |
| rs2478539 | T | G | 1 | 0.40 | 0.02 | 0.002 | 1.00E-29 | 130.32 |
| rs731024 | A | G | 1 | 0.33 | -0.01 | 0.002 | 3.10E-10 | 39.77 |
| rs488834 | T | C | 1 | 0.77 | -0.02 | 0.002 | 2.10E-19 | 78.65 |
| rs4661674 | G | A | 1 | 0.33 | -0.01 | 0.002 | 4.80E-11 | 38.10 |
| rs11210029 | G | A | 1 | 0.36 | 0.01 | 0.002 | 2.80E-10 | 37.98 |
| rs12035750 | C | T | 1 | 0.38 | 0.02 | 0.002 | 1.60E-21 | 91.83 |
| rs10776752 | T | G | 1 | 0.07 | 0.05 | 0.004 | 8.80E-34 | 143.17 |
| rs145339349 | A | G | 1 | 0.02 | 0.07 | 0.008 | 9.40E-22 | 89.31 |
| rs68085857 | T | C | 1 | 0.23 | 0.01 | 0.002 | 2.10E-08 | 27.56 |
| rs4335411 | A | G | 1 | 0.76 | 0.02 | 0.003 | 7.50E-15 | 59.17 |
| rs76099932 | A | G | 2 | 0.09 | -0.03 | 0.004 | 3.50E-18 | 70.51 |
| rs72844588 | A | G | 2 | 0.15 | 0.02 | 0.003 | 5.20E-09 | 31.46 |
| rs55732192 | T | G | 2 | 0.09 | -0.02 | 0.004 | 3.70E-11 | 43.04 |
| rs6732308 | G | A | 2 | 0.21 | -0.02 | 0.003 | 6.00E-15 | 55.97 |
| rs11900857 | C | T | 2 | 0.27 | -0.02 | 0.002 | 1.10E-12 | 48.96 |
| rs4531890 | G | A | 2 | 0.42 | 0.02 | 0.002 | 9.70E-21 | 88.90 |
| rs6716707 | G | A | 2 | 0.42 | -0.01 | 0.002 | 1.70E-10 | 39.68 |
| rs2241432 | G | A | 2 | 0.65 | 0.02 | 0.002 | 1.40E-13 | 52.65 |
| rs71427097 | T | C | 2 | 0.03 | 0.03 | 0.006 | 2.90E-08 | 29.51 |
| rs1275985 | T | C | 2 | 0.62 | -0.03 | 0.002 | 2.70E-47 | 198.13 |
| rs72792829 | T | C | 2 | 0.22 | -0.02 | 0.002 | 1.00E-16 | 72.30 |
| rs12466788 | C | T | 2 | 0.47 | 0.01 | 0.002 | 4.00E-10 | 37.76 |
| rs7593864 | A | G | 2 | 0.76 | 0.02 | 0.002 | 6.10E-19 | 79.45 |
| rs4672082 | A | G | 2 | 0.57 | -0.01 | 0.002 | 1.30E-10 | 39.74 |
| rs13020428 | G | T | 2 | 0.19 | -0.02 | 0.003 | 4.80E-12 | 47.68 |
| rs12693982 | T | C | 2 | 0.40 | 0.02 | 0.002 | 1.50E-13 | 52.19 |
| rs10804330 | C | T | 2 | 0.43 | -0.01 | 0.002 | 7.70E-13 | 50.42 |
| rs2059752 | T | C | 2 | 0.90 | -0.02 | 0.004 | 1.20E-09 | 36.66 |
| rs56190546 | A | G | 2 | 0.25 | -0.01 | 0.002 | 2.70E-08 | 27.51 |
| rs2048240 | T | C | 2 | 0.51 | 0.01 | 0.002 | 3.90E-09 | 36.54 |
| rs17406625 | C | T | 2 | 0.29 | -0.02 | 0.002 | 1.30E-13 | 53.92 |
| rs72810219 | T | C | 2 | 0.18 | 0.02 | 0.003 | 8.90E-11 | 47.46 |
| rs2249105 | G | A | 2 | 0.36 | -0.02 | 0.002 | 7.50E-22 | 91.15 |
| rs1374326 | A | G | 2 | 0.29 | 0.02 | 0.002 | 3.70E-13 | 56.40 |
| rs2571445 | G | A | 2 | 0.61 | -0.02 | 0.002 | 1.80E-14 | 58.68 |
| rs189267552 | A | T | 3 | 0.01 | -0.06 | 0.009 | 2.40E-10 | 38.06 |
| rs2306272 | C | T | 3 | 0.29 | -0.01 | 0.002 | 1.40E-09 | 34.01 |
| rs6807945 | T | C | 3 | 0.84 | -0.02 | 0.003 | 5.90E-11 | 44.15 |
| rs263017 | G | A | 3 | 0.51 | -0.01 | 0.002 | 3.20E-11 | 45.08 |
| rs4687477 | C | T | 3 | 0.39 | 0.01 | 0.002 | 6.40E-13 | 48.88 |
| rs743395 | T | C | 3 | 0.38 | 0.01 | 0.002 | 5.40E-11 | 43.06 |
| rs3772219 | C | A | 3 | 0.33 | -0.02 | 0.002 | 1.50E-13 | 50.62 |
| rs2643826 | T | C | 3 | 0.45 | 0.03 | 0.002 | 2.20E-39 | 177.26 |
| rs6768542 | A | G | 3 | 0.16 | -0.02 | 0.003 | 3.00E-10 | 40.89 |
| rs12494304 | T | C | 3 | 0.66 | 0.02 | 0.002 | 1.10E-21 | 98.13 |
| rs9841978 | A | G | 3 | 0.32 | 0.01 | 0.002 | 2.10E-12 | 46.09 |
| rs11915142 | A | G | 3 | 0.40 | 0.02 | 0.002 | 2.50E-22 | 91.08 |
| rs4679729 | T | C | 3 | 0.26 | 0.02 | 0.002 | 1.70E-10 | 41.55 |
| rs12638862 | G | A | 3 | 0.26 | -0.01 | 0.002 | 2.70E-09 | 33.31 |
| rs76508707 | T | C | 3 | 0.49 | 0.02 | 0.002 | 1.10E-15 | 63.74 |
| rs3749206 | G | A | 3 | 0.50 | 0.01 | 0.002 | 8.30E-09 | 33.43 |
| rs79692989 | T | C | 3 | 0.10 | -0.02 | 0.003 | 3.90E-12 | 46.97 |
| rs7630814 | A | G | 3 | 0.82 | 0.02 | 0.003 | 1.20E-15 | 67.01 |
| rs904475 | A | G | 3 | 0.63 | 0.02 | 0.002 | 4.10E-20 | 83.67 |
| rs34991912 | C | T | 3 | 0.58 | -0.02 | 0.002 | 1.60E-19 | 79.16 |
| rs73074830 | C | A | 3 | 0.14 | -0.02 | 0.003 | 3.50E-08 | 31.33 |
| rs11928547 | C | T | 3 | 0.41 | -0.02 | 0.002 | 3.50E-20 | 75.18 |
| rs6775484 | A | C | 3 | 0.63 | 0.01 | 0.002 | 3.20E-11 | 45.35 |
| rs2237001 | A | G | 4 | 0.13 | 0.02 | 0.003 | 1.50E-08 | 30.89 |
| rs62290561 | C | T | 4 | 0.44 | 0.01 | 0.002 | 2.90E-09 | 33.75 |
| rs11721984 | T | C | 4 | 0.46 | -0.02 | 0.002 | 1.40E-15 | 59.79 |
| rs57826934 | T | C | 4 | 0.33 | 0.01 | 0.002 | 4.60E-11 | 45.76 |
| rs7675258 | A | G | 4 | 0.54 | -0.01 | 0.002 | 7.60E-11 | 42.08 |
| rs1229984 | C | T | 4 | 0.97 | 0.05 | 0.006 | 1.70E-12 | 51.97 |
| rs13107325 | T | C | 4 | 0.08 | -0.05 | 0.004 | 1.50E-36 | 160.02 |
| rs13106448 | G | T | 4 | 0.57 | -0.02 | 0.002 | 1.60E-15 | 61.48 |
| rs6815273 | A | G | 4 | 0.43 | -0.02 | 0.002 | 8.50E-16 | 59.16 |
| rs13129779 | T | C | 4 | 0.50 | -0.01 | 0.002 | 1.90E-12 | 47.46 |
| rs979532 | T | C | 4 | 0.74 | 0.02 | 0.002 | 1.20E-12 | 47.98 |
| rs17083718 | G | A | 4 | 0.10 | -0.03 | 0.003 | 1.30E-13 | 55.07 |
| rs1847333 | T | C | 4 | 0.31 | 0.01 | 0.002 | 2.30E-08 | 31.09 |
| rs6823767 | C | T | 4 | 0.28 | 0.01 | 0.002 | 2.50E-09 | 35.33 |
| rs12643599 | G | A | 4 | 0.36 | -0.02 | 0.002 | 1.10E-22 | 96.63 |
| rs11737334 | G | A | 4 | 0.32 | 0.01 | 0.002 | 1.80E-10 | 34.79 |
| rs55924432 | T | C | 4 | 0.40 | 0.01 | 0.002 | 2.10E-12 | 48.69 |
| rs2945330 | C | A | 4 | 0.47 | -0.01 | 0.002 | 1.80E-11 | 44.19 |
| rs6600888 | T | C | 4 | 0.46 | 0.01 | 0.002 | 7.20E-09 | 32.68 |
| rs13125101 | A | G | 4 | 0.29 | 0.05 | 0.002 | 7.50E-117 | 516.15 |
| rs9992744 | C | T | 4 | 0.57 | -0.01 | 0.002 | 4.60E-08 | 30.06 |
| rs10032729 | A | G | 4 | 0.51 | 0.01 | 0.002 | 4.80E-11 | 42.72 |
| rs12508096 | C | T | 4 | 0.35 | 0.01 | 0.002 | 2.10E-08 | 31.60 |
| rs10517653 | T | G | 4 | 0.15 | -0.02 | 0.003 | 5.10E-14 | 55.63 |
| rs10069690 | T | C | 5 | 0.26 | 0.02 | 0.002 | 3.80E-12 | 48.52 |
| rs1173727 | C | T | 5 | 0.60 | 0.04 | 0.002 | 1.80E-83 | 376.06 |
| rs1644318 | C | T | 5 | 0.39 | 0.02 | 0.002 | 5.20E-21 | 87.20 |
| rs10900864 | G | A | 5 | 0.70 | 0.01 | 0.002 | 4.30E-09 | 32.32 |
| rs778593 | C | T | 5 | 0.42 | 0.02 | 0.002 | 2.20E-13 | 55.60 |
| rs59851826 | G | A | 5 | 0.29 | 0.02 | 0.002 | 2.70E-14 | 61.59 |
| rs17242380 | C | T | 5 | 0.27 | 0.02 | 0.002 | 8.20E-21 | 85.56 |
| rs7704651 | A | G | 5 | 0.33 | -0.01 | 0.002 | 9.10E-11 | 42.31 |
| rs17677603 | G | A | 5 | 0.39 | 0.02 | 0.002 | 7.40E-24 | 104.16 |
| rs72492447 | C | T | 5 | 0.05 | -0.03 | 0.005 | 6.90E-10 | 36.41 |
| rs256838 | T | C | 5 | 0.20 | -0.01 | 0.003 | 2.70E-08 | 29.36 |
| rs7701003 | G | A | 5 | 0.37 | -0.03 | 0.002 | 4.80E-33 | 140.13 |
| rs36071027 | T | C | 5 | 0.36 | -0.02 | 0.002 | 2.00E-13 | 52.58 |
| rs16892892 | G | A | 5 | 0.31 | 0.01 | 0.002 | 4.00E-09 | 34.71 |
| rs35711462 | G | A | 5 | 0.51 | -0.01 | 0.002 | 1.10E-10 | 41.70 |
| rs246978 | G | T | 5 | 0.29 | 0.02 | 0.002 | 3.40E-14 | 56.96 |
| rs6884350 | A | G | 5 | 0.47 | -0.01 | 0.002 | 2.50E-14 | 52.61 |
| rs62361338 | A | G | 5 | 0.24 | -0.02 | 0.002 | 2.90E-10 | 40.34 |
| rs2569882 | C | T | 6 | 0.44 | -0.01 | 0.002 | 6.90E-11 | 44.37 |
| rs7451008 | C | T | 6 | 0.26 | 0.01 | 0.002 | 3.10E-10 | 39.24 |
| rs11153071 | A | G | 6 | 0.19 | 0.02 | 0.003 | 1.40E-16 | 62.05 |
| rs13210511 | T | C | 6 | 0.44 | 0.03 | 0.002 | 8.40E-54 | 235.19 |
| rs7744902 | A | G | 6 | 0.08 | -0.02 | 0.004 | 4.30E-10 | 41.07 |
| rs111663960 | G | A | 6 | 0.12 | 0.03 | 0.003 | 2.70E-24 | 101.32 |
| rs10456100 | T | C | 6 | 0.28 | 0.01 | 0.002 | 2.40E-08 | 29.35 |
| rs6905288 | A | G | 6 | 0.57 | 0.01 | 0.002 | 4.10E-11 | 37.49 |
| rs1925148 | G | A | 6 | 0.56 | -0.01 | 0.002 | 6.80E-13 | 52.31 |
| rs2153960 | A | G | 6 | 0.71 | -0.01 | 0.002 | 4.30E-10 | 35.86 |
| rs62430713 | C | T | 6 | 0.18 | -0.02 | 0.003 | 2.80E-11 | 44.73 |
| rs7761133 | C | T | 6 | 0.15 | -0.01 | 0.003 | 2.60E-08 | 27.15 |
| rs1630736 | T | C | 6 | 0.47 | -0.01 | 0.002 | 2.20E-08 | 29.94 |
| rs150021957 | T | C | 6 | 0.07 | -0.03 | 0.006 | 1.90E-08 | 30.17 |
| rs1077393 | G | A | 6 | 0.48 | 0.02 | 0.003 | 2.20E-17 | 69.52 |
| rs7764523 | A | G | 6 | 0.32 | 0.02 | 0.002 | 1.60E-18 | 74.68 |
| rs9476307 | G | A | 6 | 0.41 | -0.01 | 0.002 | 1.00E-09 | 39.98 |
| rs13199610 | A | G | 6 | 0.11 | -0.02 | 0.003 | 6.10E-09 | 30.80 |
| rs434578 | T | C | 6 | 0.86 | -0.02 | 0.003 | 1.50E-15 | 64.13 |
| rs9355878 | A | G | 6 | 0.81 | -0.02 | 0.003 | 3.30E-09 | 35.58 |
| rs6926537 | A | T | 6 | 0.49 | 0.02 | 0.002 | 2.10E-15 | 60.60 |
| rs2327429 | C | T | 6 | 0.30 | -0.01 | 0.002 | 3.20E-09 | 36.71 |
| rs9266095 | G | A | 6 | 0.21 | -0.02 | 0.003 | 5.40E-11 | 39.49 |
| rs9285425 | A | G | 6 | 0.50 | -0.01 | 0.002 | 1.00E-11 | 45.66 |
| rs42038 | T | C | 7 | 0.30 | -0.02 | 0.002 | 8.50E-26 | 109.82 |
| rs12154627 | C | T | 7 | 0.50 | -0.02 | 0.002 | 6.20E-13 | 52.64 |
| rs35761891 | G | A | 7 | 0.05 | 0.04 | 0.005 | 4.20E-12 | 48.98 |
| rs2023843 | T | C | 7 | 0.93 | 0.05 | 0.004 | 1.40E-40 | 178.94 |
| rs34990159 | A | G | 7 | 0.60 | 0.02 | 0.002 | 1.10E-20 | 84.19 |
| rs10241964 | A | G | 7 | 0.10 | 0.03 | 0.003 | 1.80E-16 | 68.02 |
| rs62481856 | A | G | 7 | 0.20 | 0.05 | 0.003 | 5.00E-75 | 335.91 |
| rs12668436 | C | T | 7 | 0.25 | 0.01 | 0.002 | 9.30E-10 | 37.50 |
| rs706159 | A | G | 7 | 0.43 | -0.01 | 0.002 | 8.30E-13 | 52.04 |
| rs73033340 | G | A | 7 | 0.03 | -0.05 | 0.006 | 1.80E-17 | 76.74 |
| rs2906157 | G | A | 7 | 0.64 | -0.02 | 0.002 | 3.90E-12 | 52.17 |
| rs4507656 | G | C | 7 | 0.30 | 0.01 | 0.002 | 9.20E-10 | 35.15 |
| rs35844144 | T | C | 7 | 0.74 | -0.01 | 0.002 | 1.00E-08 | 31.74 |
| rs10215731 | C | T | 7 | 0.26 | -0.01 | 0.002 | 3.70E-09 | 30.41 |
| rs56261656 | A | G | 7 | 0.17 | -0.01 | 0.003 | 1.80E-08 | 29.41 |
| rs3918226 | T | C | 7 | 0.08 | 0.04 | 0.004 | 4.20E-24 | 105.83 |
| rs17173238 | G | A | 7 | 0.29 | 0.02 | 0.002 | 2.70E-29 | 121.45 |
| rs10261098 | T | C | 7 | 0.15 | 0.02 | 0.003 | 1.20E-08 | 32.06 |
| rs145151767 | A | G | 8 | 0.25 | 0.02 | 0.002 | 8.10E-15 | 57.26 |
| rs2978456 | C | T | 8 | 0.45 | 0.01 | 0.002 | 3.90E-13 | 50.13 |
| rs28823280 | A | G | 8 | 0.25 | -0.02 | 0.002 | 1.20E-12 | 52.91 |
| rs74900445 | C | T | 8 | 0.12 | 0.02 | 0.003 | 3.00E-13 | 50.11 |
| rs894348 | G | A | 8 | 0.40 | 0.02 | 0.002 | 1.10E-13 | 54.72 |
| rs10096038 | T | C | 8 | 0.70 | -0.01 | 0.002 | 4.00E-10 | 35.87 |
| rs3802230 | A | C | 8 | 0.54 | -0.02 | 0.002 | 4.70E-19 | 78.50 |
| rs76020419 | T | G | 8 | 0.04 | -0.03 | 0.006 | 6.80E-09 | 35.47 |
| rs2280870 | G | T | 8 | 0.27 | 0.01 | 0.002 | 1.80E-08 | 32.99 |
| rs1693567 | C | T | 8 | 0.46 | -0.02 | 0.002 | 2.20E-15 | 63.85 |
| rs28459406 | A | G | 8 | 0.18 | -0.02 | 0.003 | 5.30E-09 | 36.28 |
| rs17210179 | T | C | 8 | 0.16 | 0.02 | 0.003 | 2.50E-11 | 41.93 |
| rs13256088 | C | T | 8 | 0.55 | -0.01 | 0.002 | 1.90E-08 | 29.51 |
| rs35410017 | C | T | 8 | 0.24 | 0.02 | 0.002 | 4.30E-11 | 40.01 |
| rs73563812 | T | G | 8 | 0.24 | -0.03 | 0.002 | 5.20E-33 | 136.11 |
| rs4873492 | T | C | 8 | 0.17 | 0.02 | 0.003 | 2.10E-16 | 67.21 |
| rs2354862 | C | A | 8 | 0.36 | -0.02 | 0.002 | 1.60E-13 | 55.12 |
| rs28447194 | A | G | 8 | 0.10 | -0.02 | 0.003 | 1.50E-09 | 35.28 |
| rs111417100 | C | T | 8 | 0.11 | 0.02 | 0.003 | 1.70E-09 | 37.51 |
| rs7838781 | G | A | 8 | 0.17 | -0.02 | 0.003 | 8.20E-10 | 37.03 |
| rs12680480 | G | A | 8 | 0.69 | 0.01 | 0.002 | 2.40E-08 | 28.75 |
| rs2340586 | A | G | 8 | 0.51 | -0.01 | 0.002 | 1.70E-08 | 33.36 |
| rs3110054 | T | C | 8 | 0.61 | -0.01 | 0.002 | 2.90E-08 | 29.49 |
| rs17207512 | A | G | 8 | 0.38 | -0.01 | 0.002 | 7.70E-09 | 32.63 |
| rs1928244 | A | G | 9 | 0.15 | 0.02 | 0.003 | 6.40E-09 | 34.61 |
| rs10982007 | A | G | 9 | 0.55 | -0.01 | 0.002 | 2.60E-11 | 45.14 |
| rs1333045 | C | T | 9 | 0.52 | 0.01 | 0.002 | 3.30E-09 | 36.78 |
| rs76452347 | T | C | 9 | 0.20 | -0.02 | 0.003 | 1.50E-11 | 45.94 |
| rs72765298 | C | T | 9 | 0.13 | 0.02 | 0.003 | 7.80E-13 | 51.78 |
| rs1547287 | C | T | 9 | 0.14 | 0.02 | 0.003 | 3.40E-12 | 47.86 |
| rs7034315 | G | A | 9 | 0.86 | 0.02 | 0.003 | 8.40E-09 | 32.93 |
| rs10817007 | G | T | 9 | 0.13 | 0.03 | 0.003 | 2.50E-19 | 76.99 |
| rs112535608 | G | A | 9 | 0.06 | -0.02 | 0.004 | 1.50E-08 | 30.94 |
| rs6271 | T | C | 9 | 0.07 | -0.03 | 0.004 | 2.50E-19 | 79.02 |
| rs11145807 | G | A | 9 | 0.59 | -0.01 | 0.002 | 7.60E-12 | 46.86 |
| rs12258967 | G | C | 10 | 0.30 | -0.04 | 0.002 | 1.30E-73 | 318.43 |
| rs12264186 | T | C | 10 | 0.19 | 0.02 | 0.003 | 3.70E-11 | 44.69 |
| rs72831343 | G | T | 10 | 0.15 | -0.04 | 0.003 | 4.10E-50 | 221.05 |
| rs10995311 | G | C | 10 | 0.45 | -0.02 | 0.002 | 3.80E-21 | 85.87 |
| rs10883543 | T | G | 10 | 0.89 | 0.04 | 0.003 | 1.10E-39 | 168.52 |
| rs10883663 | C | T | 10 | 0.31 | 0.01 | 0.002 | 1.20E-08 | 31.98 |
| rs11196553 | T | C | 10 | 0.04 | 0.03 | 0.005 | 1.10E-10 | 43.54 |
| rs2782980 | C | T | 10 | 0.72 | 0.03 | 0.002 | 6.00E-33 | 145.25 |
| rs4752693 | T | C | 10 | 0.11 | 0.02 | 0.003 | 1.80E-11 | 47.25 |
| rs7912283 | A | G | 10 | 0.65 | -0.01 | 0.002 | 3.60E-09 | 34.91 |
| rs11252344 | T | C | 10 | 0.08 | -0.03 | 0.004 | 4.80E-11 | 42.51 |
| rs12779675 | C | G | 10 | 0.46 | 0.02 | 0.002 | 2.90E-14 | 58.84 |
| rs2177843 | T | C | 10 | 0.15 | 0.03 | 0.003 | 9.00E-19 | 75.86 |
| rs11001051 | A | C | 10 | 0.72 | -0.02 | 0.002 | 3.80E-11 | 44.41 |
| rs11202390 | T | G | 10 | 0.53 | -0.01 | 0.002 | 6.70E-09 | 32.24 |
| rs7100317 | A | C | 10 | 0.53 | 0.01 | 0.002 | 3.90E-10 | 38.05 |
| rs4948643 | C | T | 10 | 0.72 | -0.01 | 0.002 | 8.20E-11 | 39.12 |
| rs77413490 | T | G | 10 | 0.04 | 0.03 | 0.005 | 1.40E-08 | 34.36 |
| rs11187838 | A | G | 10 | 0.43 | -0.03 | 0.002 | 9.10E-46 | 197.27 |
| rs1133400 | G | A | 10 | 0.22 | 0.02 | 0.002 | 4.40E-10 | 44.28 |
| rs1623474 | T | C | 10 | 0.33 | 0.02 | 0.002 | 3.40E-28 | 118.08 |
| rs12258523 | C | T | 10 | 0.37 | -0.01 | 0.002 | 3.30E-08 | 29.95 |
| rs11191580 | C | T | 10 | 0.08 | -0.06 | 0.004 | 2.30E-60 | 253.80 |
| rs34250979 | A | G | 10 | 0.02 | 0.05 | 0.007 | 5.10E-11 | 41.88 |
| rs569550 | G | T | 11 | 0.39 | 0.03 | 0.002 | 5.10E-55 | 237.94 |
| rs11821781 | G | A | 11 | 0.26 | 0.02 | 0.002 | 2.90E-25 | 106.95 |
| rs16933682 | C | T | 11 | 0.15 | 0.02 | 0.003 | 1.70E-08 | 31.04 |
| rs2904315 | G | A | 11 | 0.68 | 0.01 | 0.002 | 2.80E-11 | 43.05 |
| rs2306363 | T | G | 11 | 0.21 | -0.03 | 0.003 | 1.40E-23 | 99.75 |
| rs604723 | C | T | 11 | 0.72 | 0.04 | 0.002 | 3.00E-81 | 363.04 |
| rs78799967 | T | C | 11 | 0.03 | -0.04 | 0.007 | 2.20E-08 | 30.53 |
| rs2018218 | G | A | 11 | 0.42 | -0.02 | 0.002 | 2.40E-17 | 70.91 |
| rs2289125 | C | A | 11 | 0.79 | 0.02 | 0.003 | 2.60E-09 | 36.16 |
| rs117828113 | C | T | 11 | 0.07 | -0.03 | 0.004 | 3.40E-11 | 41.05 |
| rs1470260 | C | T | 11 | 0.18 | 0.03 | 0.003 | 7.20E-33 | 138.11 |
| rs17309741 | C | T | 11 | 0.33 | -0.02 | 0.002 | 1.00E-14 | 61.20 |
| rs56788999 | C | T | 11 | 0.03 | -0.04 | 0.006 | 6.70E-11 | 43.74 |
| rs10160382 | C | T | 11 | 0.35 | 0.02 | 0.002 | 2.00E-12 | 48.54 |
| rs67885470 | T | C | 11 | 0.21 | -0.01 | 0.003 | 1.00E-08 | 32.74 |
| rs1991196 | C | T | 11 | 0.33 | -0.02 | 0.002 | 1.10E-18 | 80.13 |
| rs236916 | A | G | 11 | 0.13 | 0.02 | 0.003 | 1.60E-10 | 40.14 |
| rs4936109 | C | T | 11 | 0.67 | 0.01 | 0.002 | 5.90E-09 | 33.40 |
| rs7927974 | G | A | 11 | 0.28 | 0.02 | 0.002 | 1.30E-12 | 46.04 |
| rs7107356 | G | A | 11 | 0.51 | 0.03 | 0.002 | 8.10E-55 | 239.01 |
| rs573455 | G | A | 11 | 0.53 | -0.01 | 0.002 | 3.80E-12 | 50.27 |
| rs12809972 | T | C | 12 | 0.36 | -0.01 | 0.002 | 1.90E-08 | 30.07 |
| rs4763297 | C | A | 12 | 0.43 | 0.02 | 0.002 | 3.40E-15 | 58.39 |
| rs7970350 | T | C | 12 | 0.49 | 0.02 | 0.002 | 1.20E-13 | 55.82 |
| rs1896326 | A | G | 12 | 0.23 | -0.02 | 0.002 | 7.30E-10 | 37.13 |
| rs35427 | G | T | 12 | 0.38 | -0.03 | 0.002 | 4.50E-38 | 163.67 |
| rs11834380 | A | C | 12 | 0.10 | -0.02 | 0.003 | 4.40E-09 | 34.68 |
| rs7134440 | T | C | 12 | 0.08 | 0.03 | 0.004 | 3.40E-14 | 60.81 |
| rs7137828 | T | C | 12 | 0.52 | -0.03 | 0.002 | 3.50E-58 | 247.44 |
| rs61917655 | T | C | 12 | 0.09 | 0.02 | 0.004 | 7.10E-12 | 43.99 |
| rs12579302 | G | A | 12 | 0.17 | -0.05 | 0.003 | 5.80E-71 | 312.29 |
| rs76895963 | G | T | 12 | 0.02 | -0.05 | 0.008 | 1.10E-11 | 43.31 |
| rs60691990 | C | T | 12 | 0.34 | -0.02 | 0.002 | 5.00E-31 | 127.32 |
| rs1480043 | C | T | 12 | 0.50 | 0.01 | 0.002 | 2.80E-08 | 30.10 |
| rs6539344 | T | G | 12 | 0.57 | 0.01 | 0.002 | 7.00E-13 | 50.44 |
| rs4767332 | A | C | 12 | 0.58 | 0.02 | 0.002 | 6.00E-17 | 67.75 |
| rs11615755 | G | T | 12 | 0.48 | 0.01 | 0.002 | 3.50E-10 | 37.54 |
| rs10771962 | T | G | 12 | 0.35 | 0.01 | 0.002 | 8.70E-09 | 32.00 |
| rs4883481 | C | T | 12 | 0.63 | -0.02 | 0.002 | 3.50E-23 | 92.50 |
| rs10876531 | C | A | 12 | 0.29 | -0.02 | 0.002 | 9.70E-26 | 109.13 |
| rs1169078 | G | C | 12 | 0.31 | 0.01 | 0.002 | 1.10E-09 | 34.43 |
| rs2289901 | T | C | 13 | 0.15 | 0.02 | 0.003 | 9.80E-10 | 36.87 |
| rs10454590 | C | T | 13 | 0.10 | 0.02 | 0.003 | 9.20E-10 | 37.03 |
| rs606950 | A | G | 13 | 0.62 | 0.02 | 0.002 | 1.80E-19 | 79.17 |
| rs9512592 | G | C | 13 | 0.11 | -0.02 | 0.003 | 2.60E-08 | 30.84 |
| rs78838977 | G | A | 13 | 0.07 | -0.03 | 0.004 | 7.00E-11 | 41.63 |
| rs17245822 | C | A | 13 | 0.37 | 0.01 | 0.002 | 1.10E-08 | 37.57 |
| rs1807797 | A | G | 13 | 0.39 | -0.01 | 0.002 | 1.80E-09 | 36.05 |
| rs9549328 | T | C | 13 | 0.23 | 0.02 | 0.002 | 1.20E-15 | 62.37 |
| rs9590403 | T | C | 13 | 0.23 | 0.02 | 0.002 | 1.20E-21 | 89.83 |
| rs8002127 | T | C | 13 | 0.95 | -0.03 | 0.005 | 5.80E-09 | 35.87 |
| rs11629120 | C | T | 14 | 0.41 | 0.01 | 0.002 | 4.40E-09 | 31.71 |
| rs8904 | A | G | 14 | 0.36 | 0.02 | 0.002 | 4.80E-14 | 51.59 |
| rs12885878 | G | A | 14 | 0.77 | 0.01 | 0.003 | 2.50E-08 | 31.97 |
| rs12050260 | C | T | 14 | 0.65 | -0.01 | 0.002 | 1.10E-12 | 46.73 |
| rs2238280 | C | T | 14 | 0.76 | 0.01 | 0.002 | 3.50E-09 | 34.77 |
| rs2236262 | G | A | 14 | 0.50 | -0.01 | 0.002 | 3.60E-11 | 50.33 |
| rs17562391 | T | C | 14 | 0.41 | 0.01 | 0.002 | 5.60E-10 | 36.66 |
| rs72677847 | C | T | 14 | 0.04 | -0.04 | 0.006 | 2.20E-13 | 54.26 |
| rs3015455 | A | G | 14 | 0.72 | -0.01 | 0.002 | 3.70E-10 | 36.74 |
| rs76891117 | G | A | 14 | 0.10 | 0.02 | 0.003 | 8.00E-11 | 41.82 |
| rs1866628 | T | C | 14 | 0.48 | 0.01 | 0.002 | 6.90E-09 | 28.86 |
| rs28929474 | T | C | 14 | 0.02 | -0.04 | 0.007 | 1.40E-08 | 30.70 |
| rs28866311 | G | T | 15 | 0.48 | 0.02 | 0.002 | 2.80E-17 | 72.66 |
| rs2899463 | C | T | 15 | 0.51 | -0.02 | 0.002 | 4.30E-14 | 55.04 |
| rs2652813 | A | G | 15 | 0.77 | -0.02 | 0.002 | 1.60E-11 | 42.71 |
| rs2062316 | G | A | 15 | 0.44 | 0.02 | 0.002 | 2.70E-24 | 102.74 |
| rs149453951 | T | C | 15 | 0.02 | -0.07 | 0.007 | 3.90E-22 | 88.15 |
| rs10852034 | T | C | 15 | 0.41 | -0.01 | 0.002 | 5.30E-11 | 40.00 |
| rs2246754 | T | C | 15 | 0.21 | 0.01 | 0.003 | 8.40E-09 | 30.88 |
| rs3736290 | A | C | 15 | 0.53 | 0.02 | 0.002 | 8.20E-19 | 77.18 |
| rs11636952 | C | T | 15 | 0.69 | -0.03 | 0.002 | 7.80E-43 | 180.11 |
| rs11076856 | C | T | 16 | 0.57 | 0.02 | 0.002 | 3.00E-20 | 80.84 |
| rs7404754 | T | C | 16 | 0.57 | -0.01 | 0.002 | 2.30E-10 | 37.32 |
| rs2281226 | C | A | 16 | 0.60 | -0.01 | 0.002 | 1.80E-11 | 45.05 |
| rs12596630 | T | C | 16 | 0.09 | 0.03 | 0.004 | 3.80E-13 | 50.78 |
| rs35476050 | A | G | 16 | 0.02 | 0.05 | 0.008 | 8.00E-10 | 39.34 |
| rs77924615 | A | G | 16 | 0.20 | -0.03 | 0.003 | 4.20E-26 | 106.01 |
| rs35300112 | C | A | 16 | 0.25 | -0.02 | 0.002 | 6.50E-15 | 65.96 |
| rs72792102 | G | A | 16 | 0.04 | 0.03 | 0.005 | 1.90E-09 | 35.88 |
| rs8044992 | C | T | 16 | 0.29 | -0.01 | 0.002 | 9.90E-10 | 37.63 |
| rs2720417 | G | A | 16 | 0.25 | 0.01 | 0.002 | 1.30E-08 | 34.80 |
| rs11641308 | C | T | 16 | 0.65 | 0.02 | 0.002 | 1.70E-21 | 96.80 |
| rs7198817 | A | C | 16 | 0.64 | -0.01 | 0.002 | 6.70E-10 | 36.11 |
| rs7186298 | T | C | 16 | 0.43 | -0.02 | 0.002 | 6.20E-13 | 53.36 |
| rs2055726 | A | G | 16 | 0.56 | 0.01 | 0.002 | 6.90E-12 | 48.49 |
| rs77870048 | T | C | 16 | 0.05 | 0.05 | 0.005 | 1.30E-31 | 132.95 |
| rs35224044 | T | C | 17 | 0.58 | 0.02 | 0.002 | 5.30E-15 | 59.28 |
| rs78744936 | A | G | 17 | 0.27 | 0.02 | 0.002 | 3.20E-15 | 62.15 |
| rs166840 | A | G | 17 | 0.41 | 0.01 | 0.002 | 6.50E-09 | 35.85 |
| rs12948326 | G | T | 17 | 0.35 | 0.03 | 0.002 | 8.40E-34 | 146.61 |
| rs55938136 | G | A | 17 | 0.22 | -0.02 | 0.002 | 3.40E-10 | 39.15 |
| rs111942199 | T | C | 17 | 0.11 | 0.02 | 0.003 | 8.60E-09 | 34.43 |
| rs6504213 | C | T | 17 | 0.59 | 0.02 | 0.002 | 6.40E-23 | 95.82 |
| rs4480845 | C | T | 17 | 0.64 | -0.02 | 0.002 | 3.20E-26 | 107.47 |
| rs62075585 | A | G | 17 | 0.53 | -0.02 | 0.002 | 2.90E-14 | 58.47 |
| rs1436138 | G | A | 17 | 0.36 | -0.02 | 0.002 | 2.10E-15 | 62.31 |
| rs62059712 | C | T | 17 | 0.08 | -0.03 | 0.004 | 1.40E-14 | 57.00 |
| rs4794923 | A | G | 17 | 0.62 | -0.01 | 0.002 | 2.00E-08 | 32.02 |
| rs17608766 | C | T | 17 | 0.15 | 0.04 | 0.003 | 1.40E-48 | 204.78 |
| rs2286526 | T | C | 17 | 0.72 | 0.02 | 0.002 | 1.00E-20 | 84.24 |
| rs3935506 | A | G | 17 | 0.24 | 0.02 | 0.002 | 1.20E-11 | 41.12 |
| rs56011283 | A | G | 17 | 0.06 | -0.03 | 0.004 | 9.40E-15 | 60.23 |
| rs66563455 | C | T | 18 | 0.18 | -0.02 | 0.003 | 3.20E-16 | 71.72 |
| rs11082394 | T | C | 18 | 0.32 | -0.02 | 0.002 | 1.50E-18 | 76.92 |
| rs12457805 | A | G | 18 | 0.30 | 0.02 | 0.002 | 5.90E-13 | 54.06 |
| rs7236548 | A | C | 18 | 0.18 | 0.02 | 0.003 | 9.70E-17 | 68.60 |
| rs1898165 | A | G | 18 | 0.52 | -0.01 | 0.002 | 7.00E-11 | 40.50 |
| rs487990 | A | G | 18 | 0.33 | 0.01 | 0.002 | 2.50E-09 | 36.79 |
| rs10048404 | T | C | 18 | 0.37 | -0.02 | 0.002 | 2.10E-13 | 52.63 |
| rs117030929 | A | G | 19 | 0.04 | 0.04 | 0.005 | 4.30E-15 | 57.69 |
| rs33842 | G | A | 19 | 0.57 | 0.01 | 0.002 | 1.30E-09 | 37.24 |
| rs17356664 | T | C | 19 | 0.31 | 0.01 | 0.002 | 4.30E-09 | 34.39 |
| rs12985940 | C | T | 19 | 0.17 | -0.03 | 0.003 | 9.00E-23 | 97.81 |
| rs12461874 | A | C | 19 | 0.28 | 0.01 | 0.002 | 7.60E-11 | 42.06 |
| rs8102876 | T | C | 19 | 0.35 | -0.02 | 0.002 | 2.10E-13 | 57.82 |
| rs439523 | C | T | 19 | 0.53 | 0.02 | 0.002 | 1.30E-16 | 71.45 |
| rs35717293 | G | A | 19 | 0.57 | 0.01 | 0.002 | 1.60E-10 | 38.13 |
| rs7412 | T | C | 19 | 0.08 | -0.04 | 0.004 | 4.70E-21 | 89.23 |
| rs118081085 | T | C | 19 | 0.04 | 0.03 | 0.005 | 1.50E-08 | 34.78 |
| rs167479 | T | G | 19 | 0.47 | -0.03 | 0.002 | 5.60E-64 | 279.52 |
| rs2032905 | G | A | 19 | 0.59 | -0.02 | 0.002 | 5.40E-18 | 74.81 |
| rs10417470 | G | A | 19 | 0.32 | -0.01 | 0.002 | 7.50E-10 | 38.56 |
| rs6072407 | A | G | 20 | 0.34 | -0.01 | 0.002 | 4.40E-11 | 39.90 |
| rs6031431 | G | A | 20 | 0.46 | 0.02 | 0.002 | 1.40E-13 | 54.41 |
| rs80346118 | A | G | 20 | 0.15 | -0.02 | 0.003 | 1.80E-18 | 72.03 |
| rs8126001 | T | C | 20 | 0.49 | -0.02 | 0.002 | 1.70E-16 | 66.53 |
| rs6054183 | C | T | 20 | 0.61 | 0.02 | 0.002 | 1.60E-14 | 57.30 |
| rs2423514 | G | A | 20 | 0.46 | -0.02 | 0.002 | 2.00E-25 | 100.24 |
| rs6141766 | G | A | 20 | 0.15 | 0.02 | 0.003 | 1.60E-15 | 62.24 |
| rs73306860 | A | G | 20 | 0.11 | 0.05 | 0.003 | 1.10E-42 | 185.90 |
| rs6108787 | G | T | 20 | 0.48 | 0.03 | 0.002 | 4.70E-35 | 153.35 |
| rs6058088 | G | T | 20 | 0.15 | -0.02 | 0.003 | 3.50E-08 | 27.16 |
| rs28572127 | C | A | 20 | 0.41 | 0.01 | 0.002 | 1.30E-09 | 32.99 |
| rs62218223 | A | G | 21 | 0.22 | 0.01 | 0.002 | 1.40E-08 | 32.03 |
| rs56072350 | A | G | 21 | 0.27 | 0.02 | 0.002 | 5.80E-12 | 45.03 |
| rs137923903 | T | C | 21 | 0.01 | -0.08 | 0.009 | 1.40E-18 | 73.90 |
| rs139919 | C | T | 22 | 0.18 | 0.02 | 0.003 | 1.30E-08 | 36.07 |
| rs1034565 | T | C | 22 | 0.28 | 0.02 | 0.002 | 3.40E-12 | 43.60 |
| rs138834 | C | A | 22 | 0.19 | -0.01 | 0.003 | 1.50E-08 | 32.07 |

**Supplementary table 2. genetic variants used as instrumental variables in the restricted analysis.**

| SNP | Effect Allele | Other Allele | Chr | EAF | Beta | SE | p-value |
| --- | --- | --- | --- | --- | --- | --- | --- |
| rs181375328 | A | G | 1 | 0.02 | -0.05 | 0.01 | 4.30E-09 |
| rs35479618 | A | G | 1 | 0.02 | 0.08 | 0.01 | 8.80E-24 |
| rs2493296 | T | C | 1 | 0.14 | 0.03 | 0.00 | 2.80E-21 |
| rs10776752 | T | G | 1 | 0.07 | 0.05 | 0.00 | 8.80E-34 |
| rs145339349 | A | G | 1 | 0.02 | 0.07 | 0.01 | 9.40E-22 |
| rs76099932 | A | G | 2 | 0.09 | -0.03 | 0.00 | 3.50E-18 |
| rs17083718 | G | A | 4 | 0.10 | -0.03 | 0.00 | 1.30E-13 |
| rs72492447 | C | T | 5 | 0.05 | -0.03 | 0.00 | 6.90E-10 |
| rs7701003 | G | A | 5 | 0.37 | -0.03 | 0.00 | 4.80E-33 |
| rs111663960 | G | A | 6 | 0.12 | 0.03 | 0.00 | 2.70E-24 |
| rs35761891 | G | A | 7 | 0.05 | 0.04 | 0.01 | 4.20E-12 |
| rs10241964 | A | G | 7 | 0.10 | 0.03 | 0.00 | 1.80E-16 |
| rs62481856 | A | G | 7 | 0.20 | 0.05 | 0.00 | 5.00E-75 |
| rs73033340 | G | A | 7 | 0.03 | -0.05 | 0.01 | 1.80E-17 |
| rs3918226 | T | C | 7 | 0.08 | 0.04 | 0.00 | 4.20E-24 |
| rs76020419 | T | G | 8 | 0.04 | -0.03 | 0.01 | 6.80E-09 |
| rs73563812 | T | G | 8 | 0.24 | -0.03 | 0.00 | 5.20E-33 |
| rs10817007 | G | T | 9 | 0.13 | 0.03 | 0.00 | 2.50E-19 |
| rs11196553 | T | C | 10 | 0.04 | 0.03 | 0.01 | 1.10E-10 |
| rs2782980 | C | T | 10 | 0.72 | 0.03 | 0.00 | 6.00E-33 |
| rs11252344 | T | C | 10 | 0.08 | -0.03 | 0.00 | 4.80E-11 |
| rs77413490 | T | G | 10 | 0.04 | 0.03 | 0.01 | 1.40E-08 |
| rs34250979 | A | G | 10 | 0.02 | 0.05 | 0.01 | 5.10E-11 |
| rs2306363 | T | G | 11 | 0.21 | -0.03 | 0.00 | 1.40E-23 |
| rs604723 | C | T | 11 | 0.72 | 0.04 | 0.00 | 3.00E-81 |
| rs78799967 | T | C | 11 | 0.03 | -0.04 | 0.01 | 2.20E-08 |
| rs117828113 | C | T | 11 | 0.07 | -0.03 | 0.00 | 3.40E-11 |
| rs1470260 | C | T | 11 | 0.18 | 0.03 | 0.00 | 7.20E-33 |
| rs56788999 | C | T | 11 | 0.03 | -0.04 | 0.01 | 6.70E-11 |
| rs78838977 | G | A | 13 | 0.07 | -0.03 | 0.00 | 7.00E-11 |
| rs8002127 | T | C | 13 | 0.95 | -0.03 | 0.00 | 5.80E-09 |
| rs149453951 | T | C | 15 | 0.02 | -0.07 | 0.01 | 3.90E-22 |
| rs35476050 | A | G | 16 | 0.02 | 0.05 | 0.01 | 8.00E-10 |
| rs62059712 | C | T | 17 | 0.08 | -0.03 | 0.00 | 1.40E-14 |
| rs17608766 | C | T | 17 | 0.15 | 0.04 | 0.00 | 1.40E-48 |
| rs117030929 | A | G | 19 | 0.04 | 0.04 | 0.01 | 4.30E-15 |
| rs12985940 | C | T | 19 | 0.17 | -0.03 | 0.00 | 9.00E-23 |
| rs118081085 | T | C | 19 | 0.04 | 0.03 | 0.01 | 1.50E-08 |
| rs167479 | T | G | 19 | 0.47 | -0.03 | 0.00 | 5.60E-64 |
| rs73306860 | A | G | 20 | 0.11 | 0.05 | 0.00 | 1.10E-42 |
| rs6108787 | G | T | 20 | 0.48 | 0.03 | 0.00 | 4.70E-35 |
| rs137923903 | T | C | 21 | 0.01 | -0.08 | 0.01 | 1.40E-18 |

**Supplementary table 3. Confounders associated with SNPs in the blue and grey clusters**

| Grey cluster | Sex hormones measurement |
| --- | --- |
|  | Mathematical ability, educational attainment |
|  | Alcohol drinking |
|  | Health span, longevity, parental longevity |
|  | Forced expiratory volume |
|  | |
| Blue cluster | Alcohol drinking |
|  | Physical activity |
|  | Asthma, endometriosis |
|  | Parental longevity |
|  | Coffee consumption |
|  | Smoking |
|  | White blood cell count |
|  | Appendicular lean mass |
|  | ADHD, autism |
|  | BMI, waist-hip ratio, type 2 diabetes |
|  | Lipid traits i.e. cholesterol, triglycerides |
|  | |
| Pink (null) cluster | Body size measures (i.e. hip circumference) |
|  | Anxiety |
|  | Sex hormone measurement |
|  | Blood lipid traits (i.e. cholesterol) |
|  | Heel bone density |
|  | Smoking |
|  | White blood cell count |

**Supplementary figures**

**Supplementary figure 1 scatter plots of SBP and breast cancer sub-types**

1.
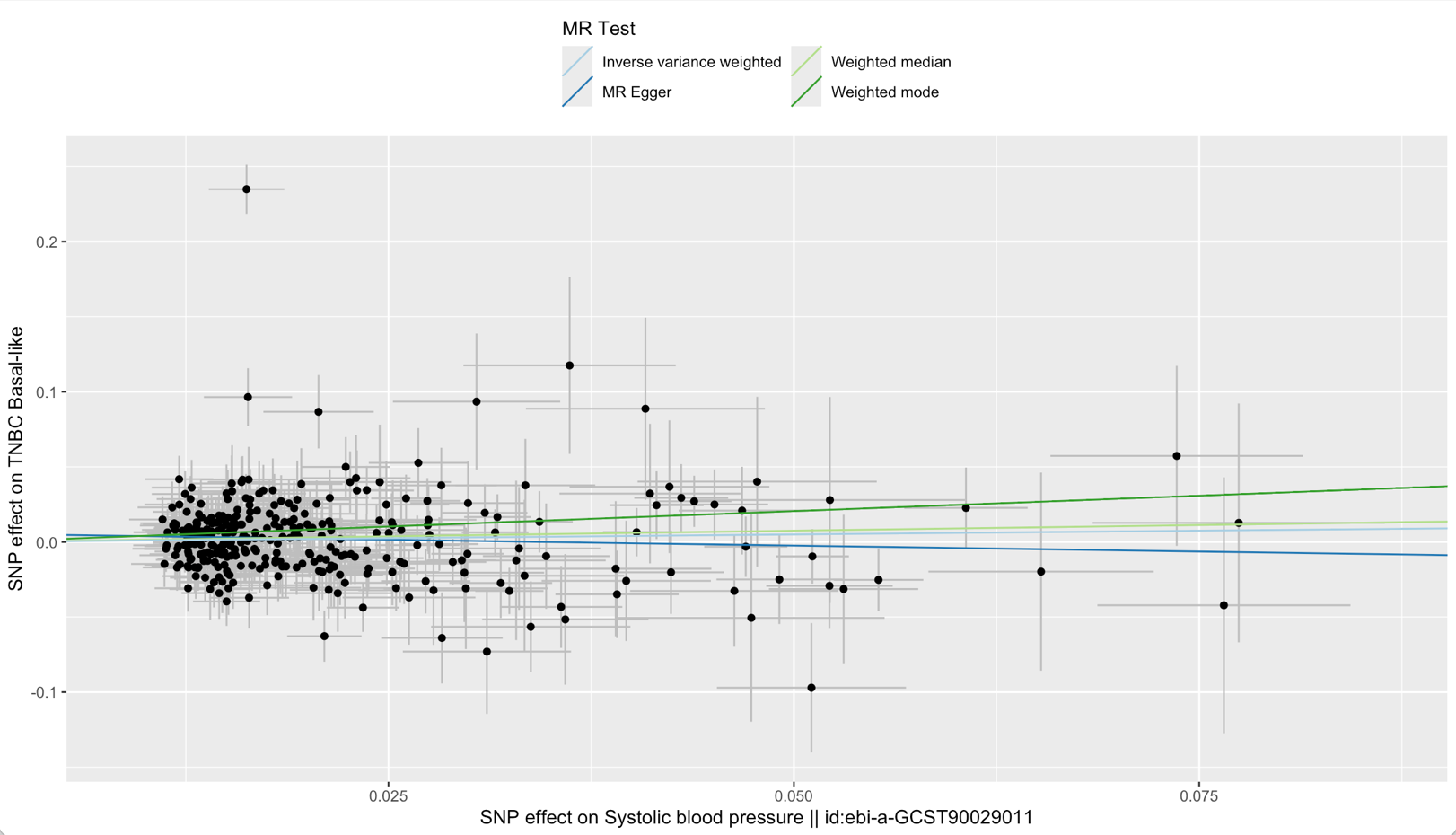
**
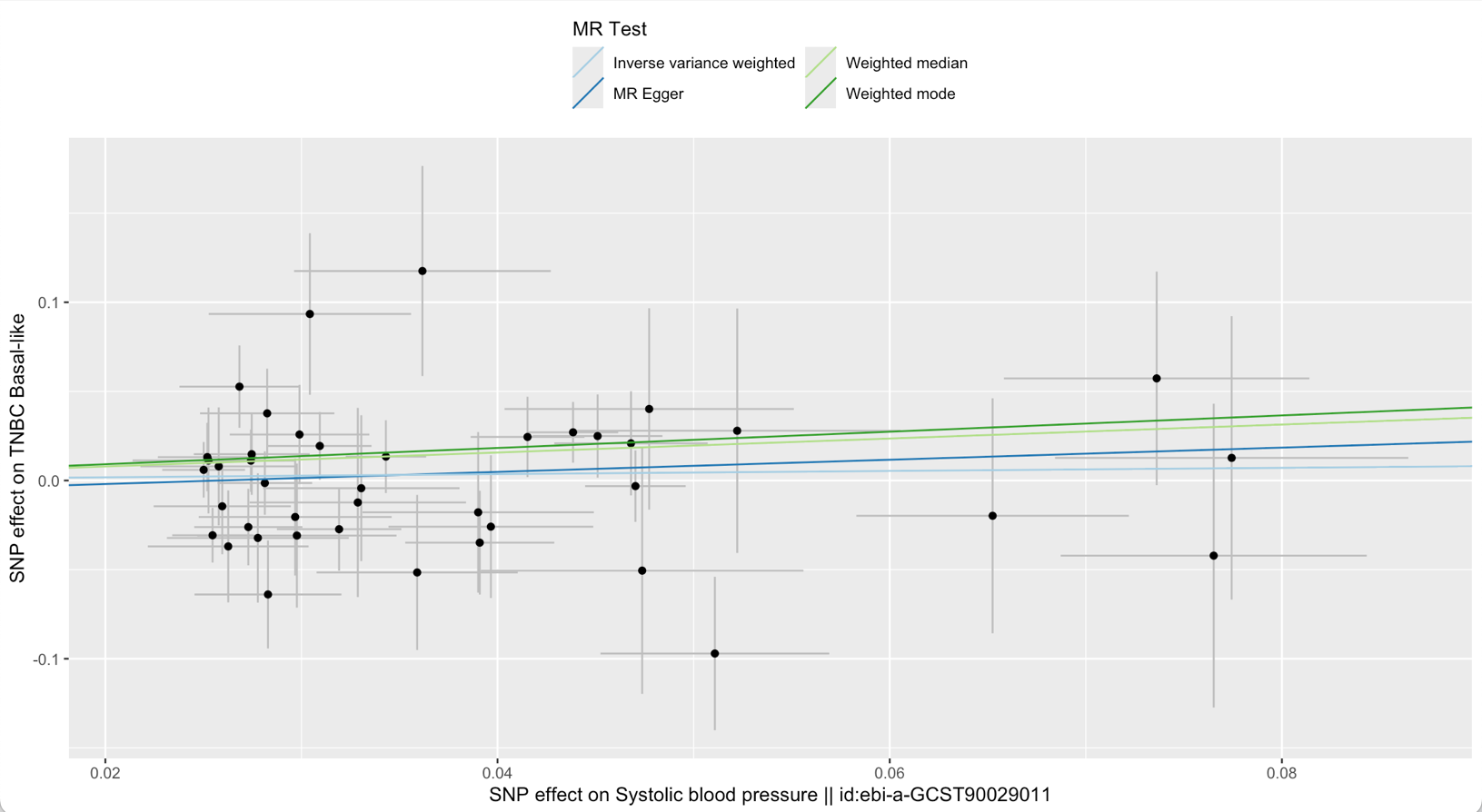
**TNBC (334 SNPs)
2. TNBC (54 SNPs)


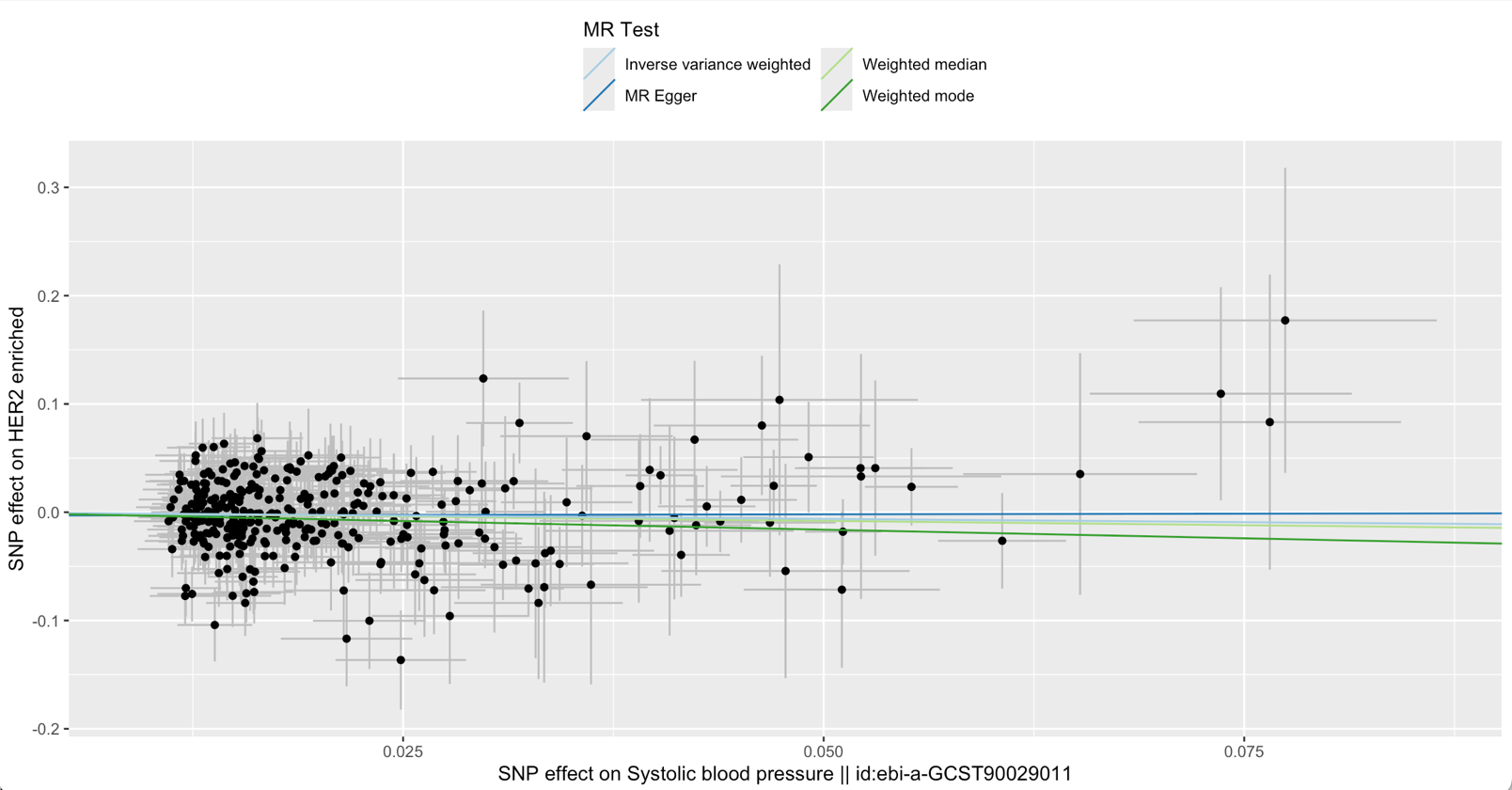


1. HER2 (334 SNPs)


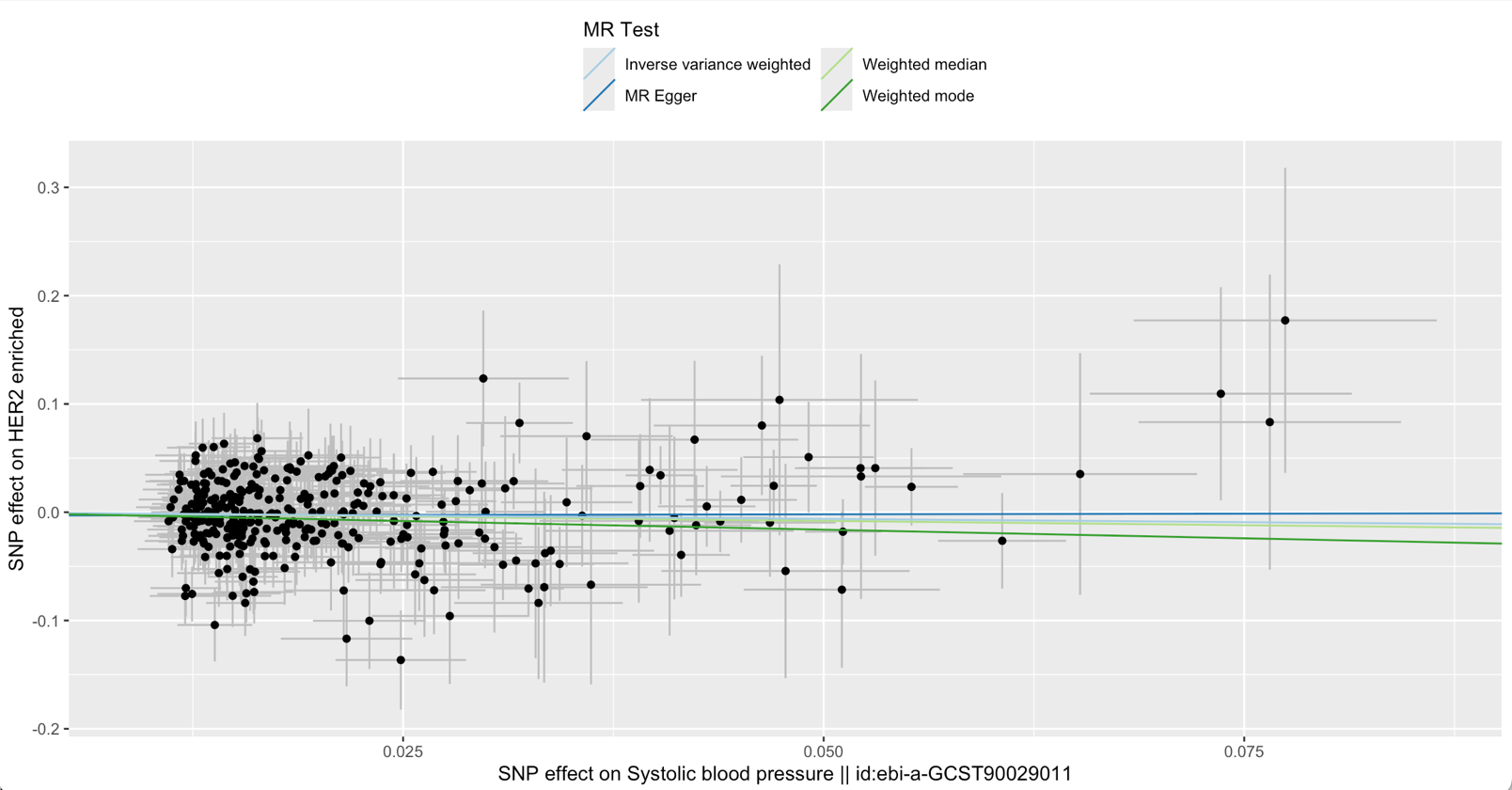


1. HER2 (54 SNPs)


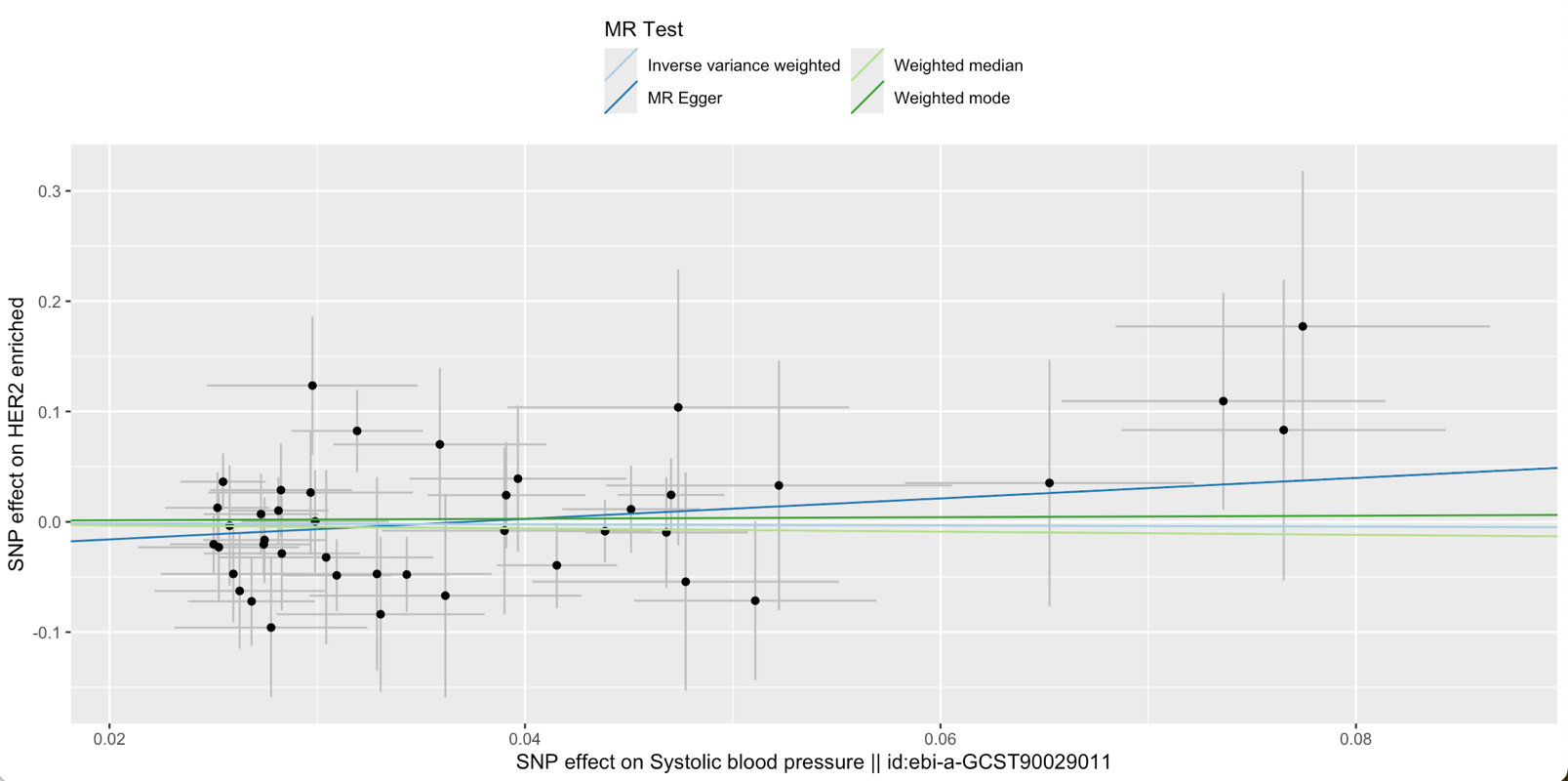


1. Luminal-A (334 SNPs)


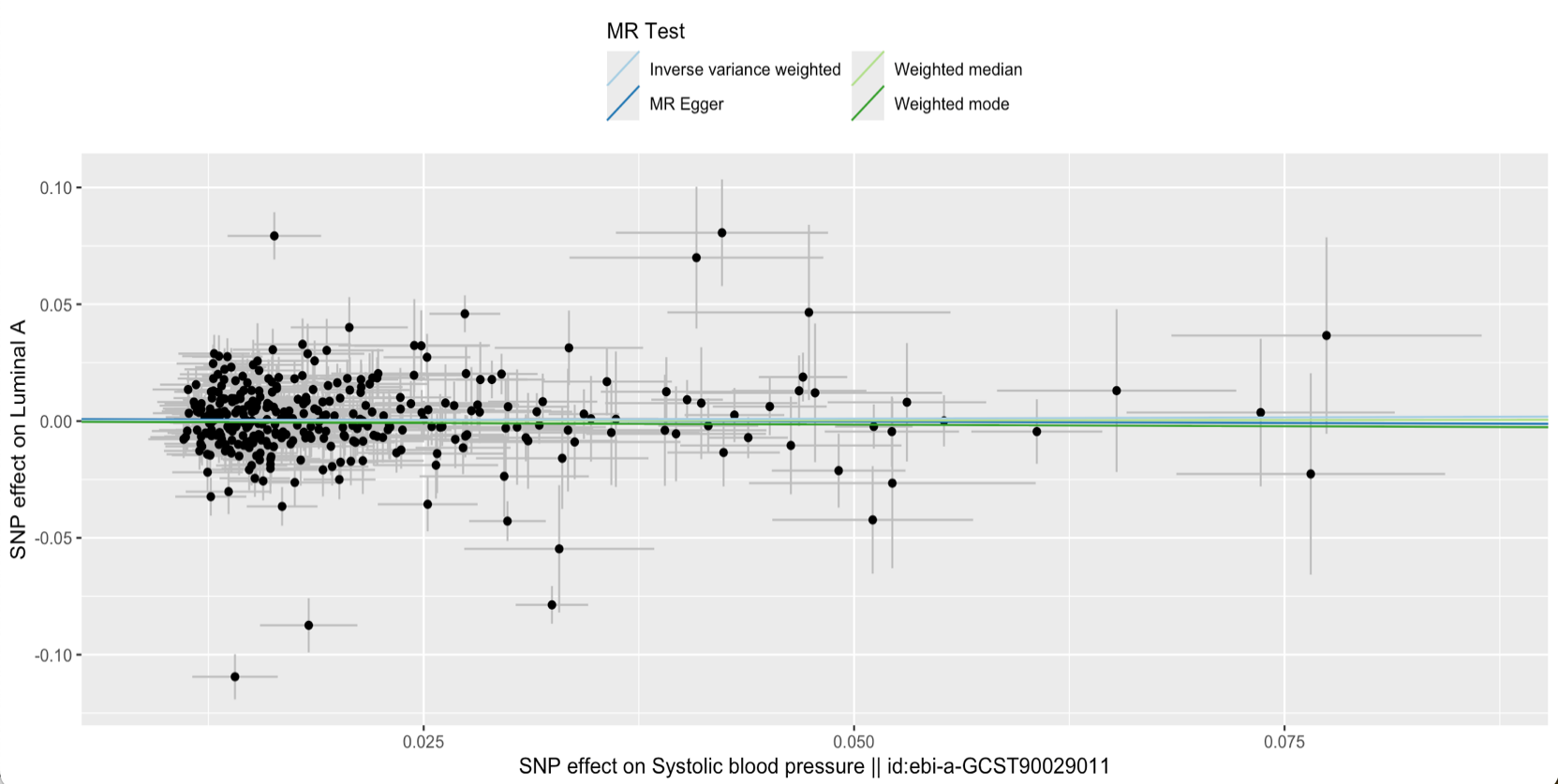


1. Luminal-A (54 SNPs)


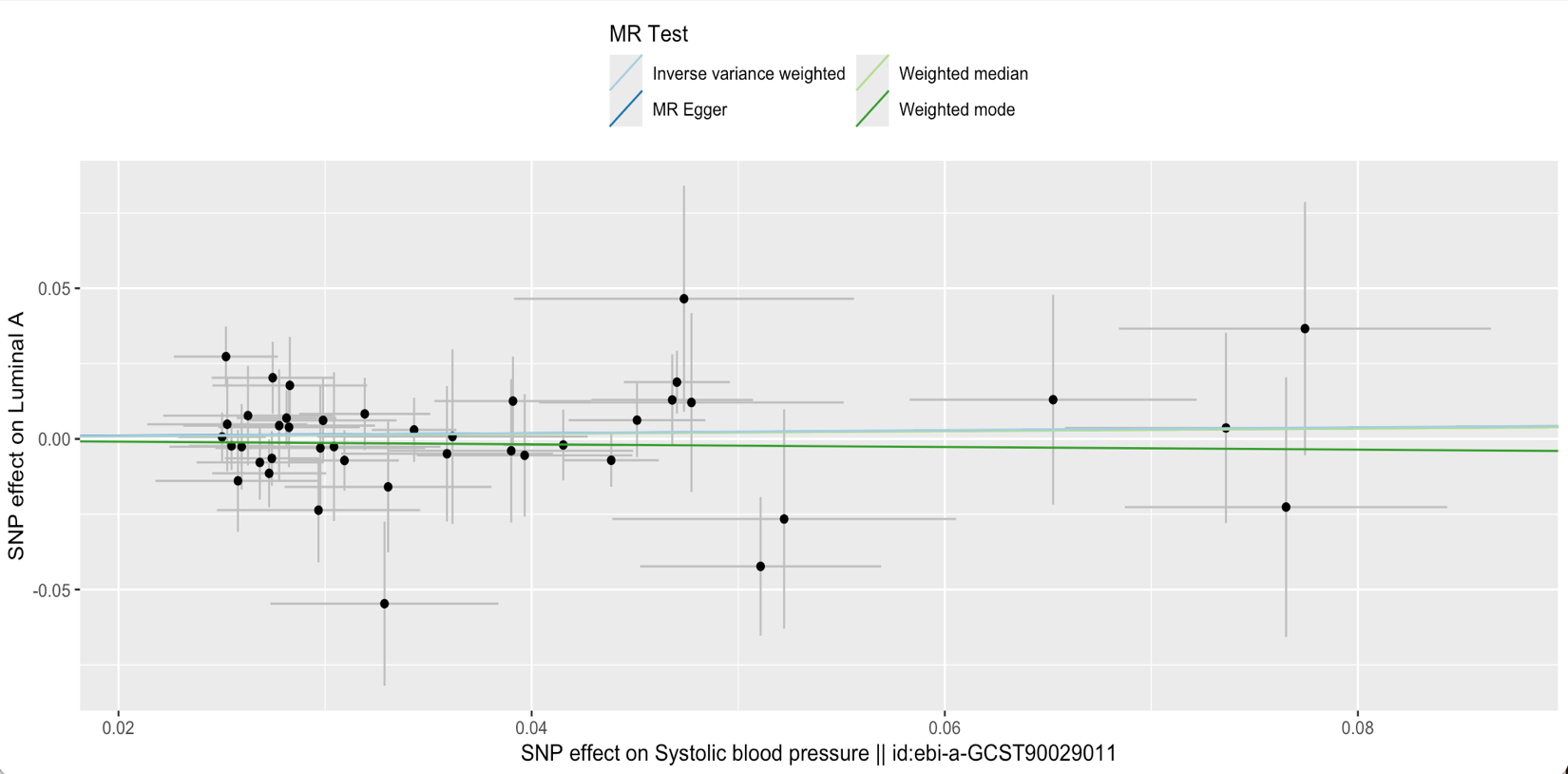


1. Luminal-B (334 SNPs)


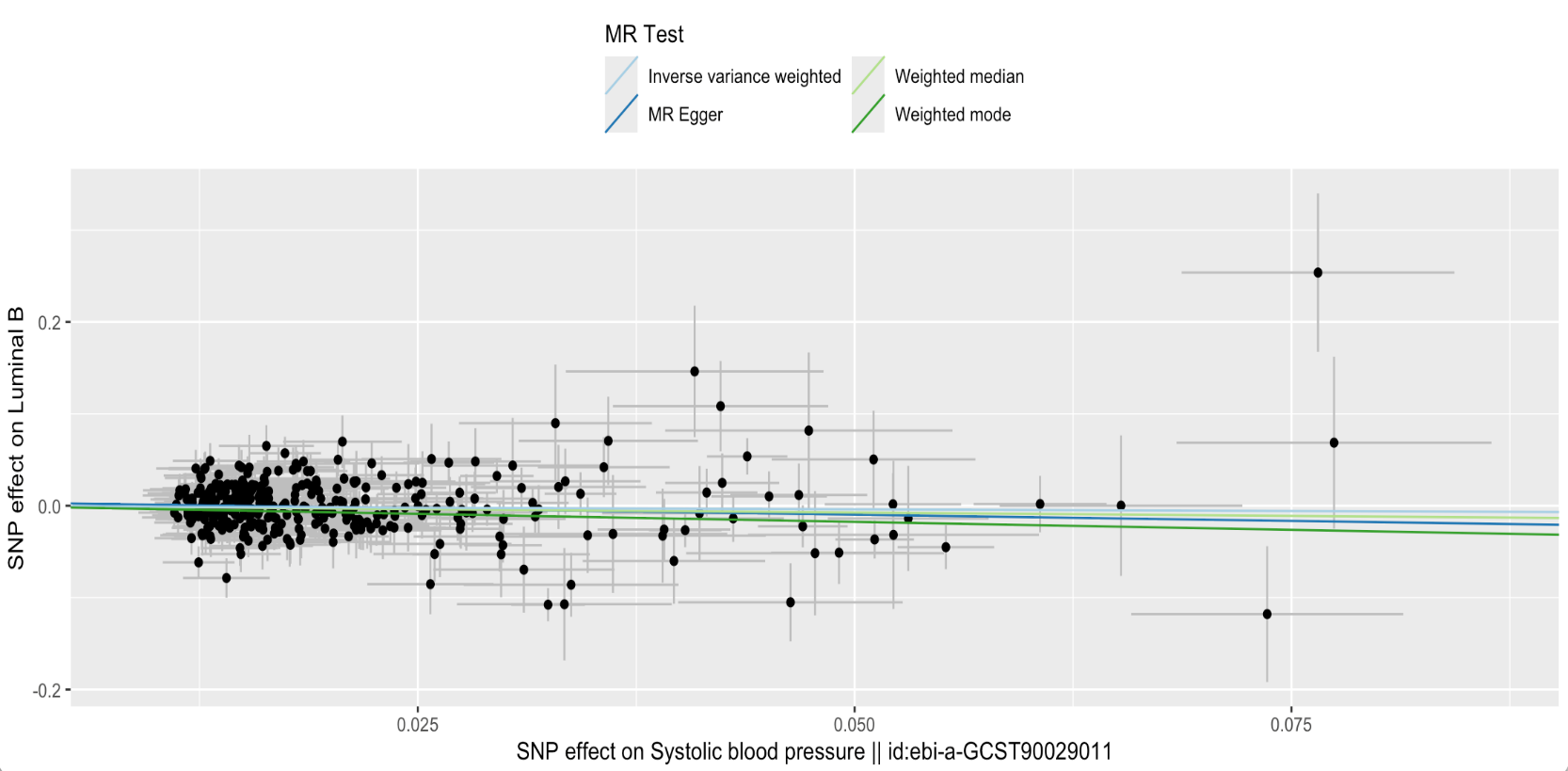


1. Luminal-B (54 SNPs)


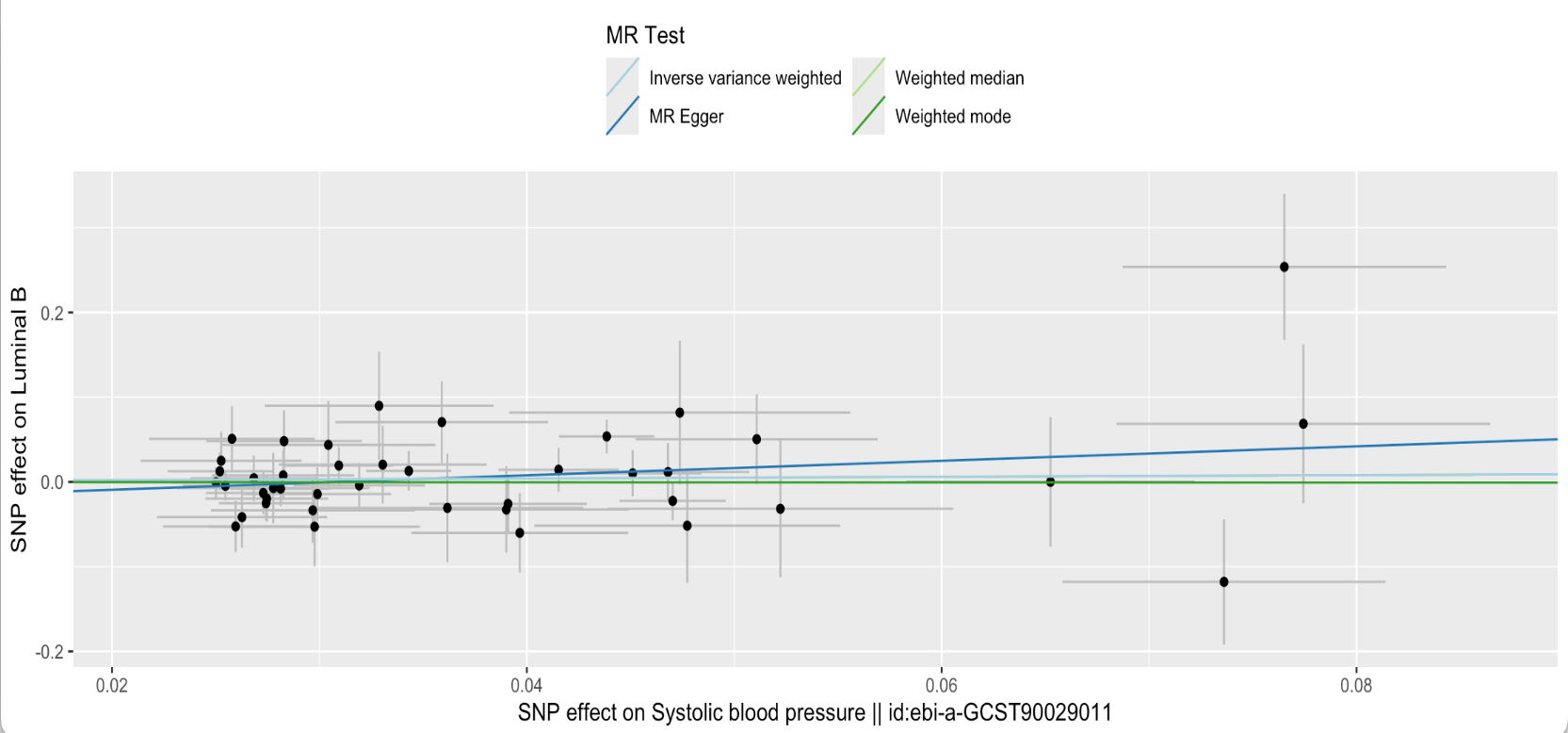


1. Luminal-B HER2 negative (334 SNPs)


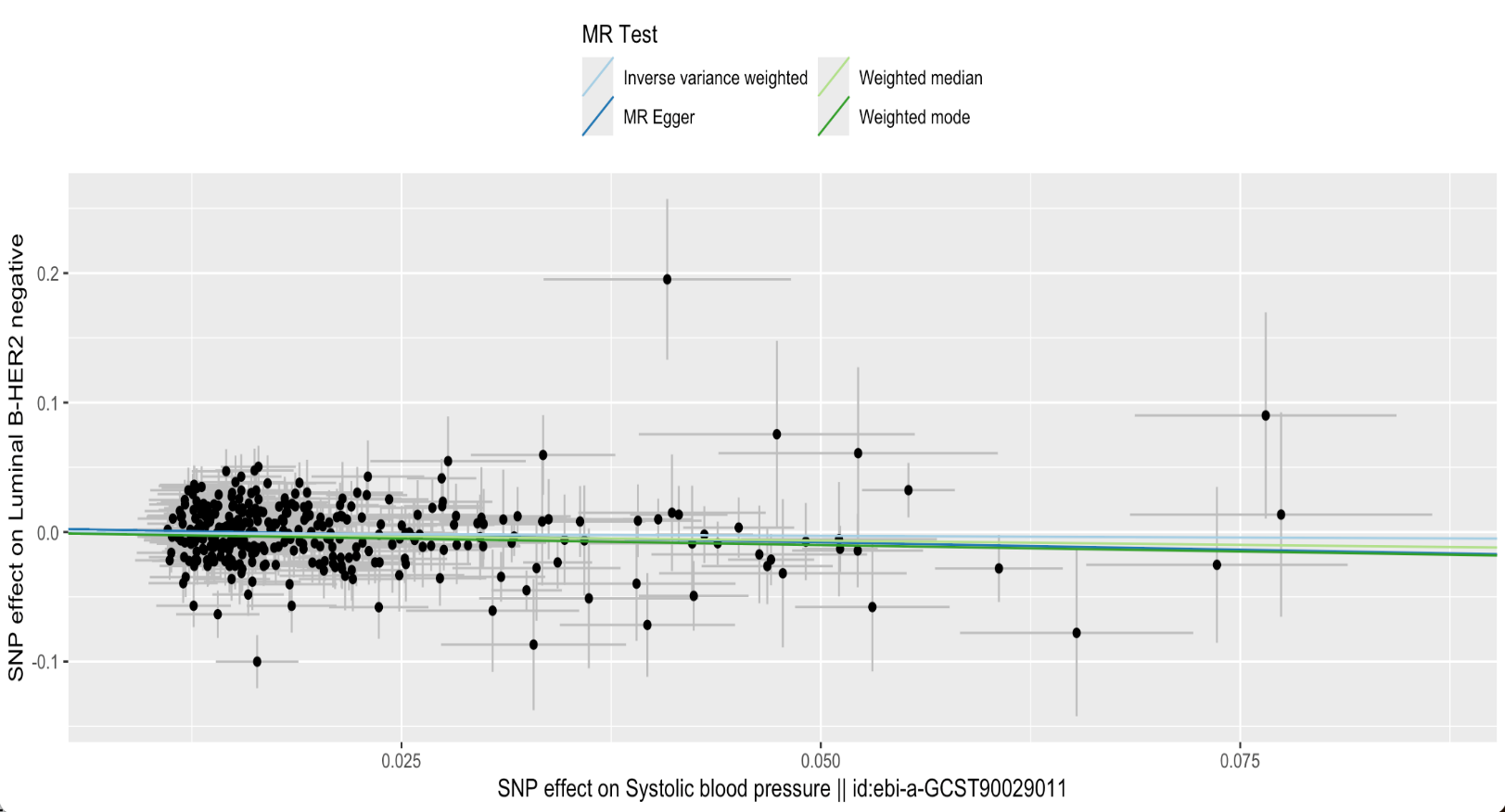


1.
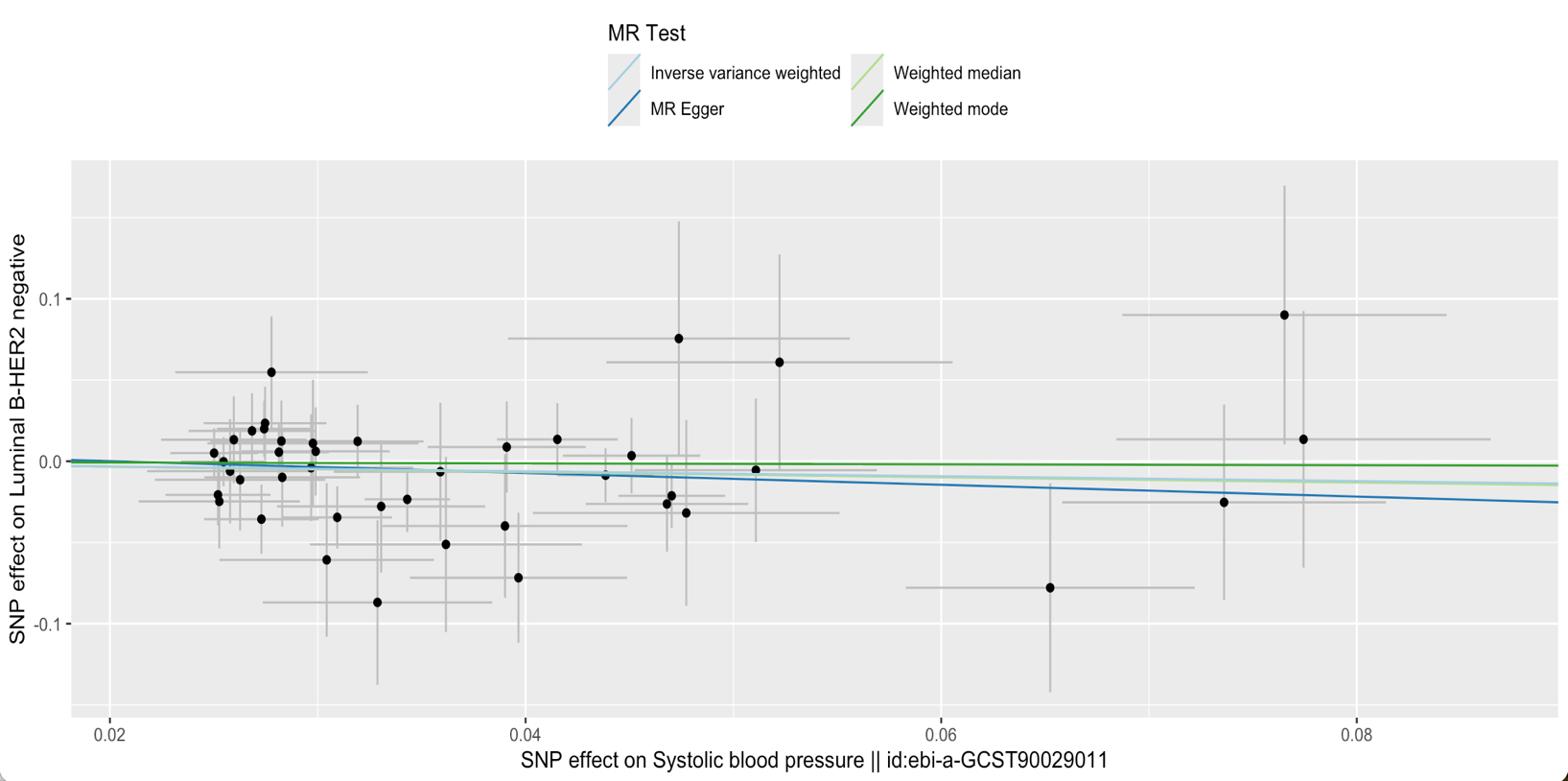
Luminal-B HER2 negative (54 SNPs)

**Supplementary figure 2. Leave-one-out plots for SBP on BC sub-types**

1.
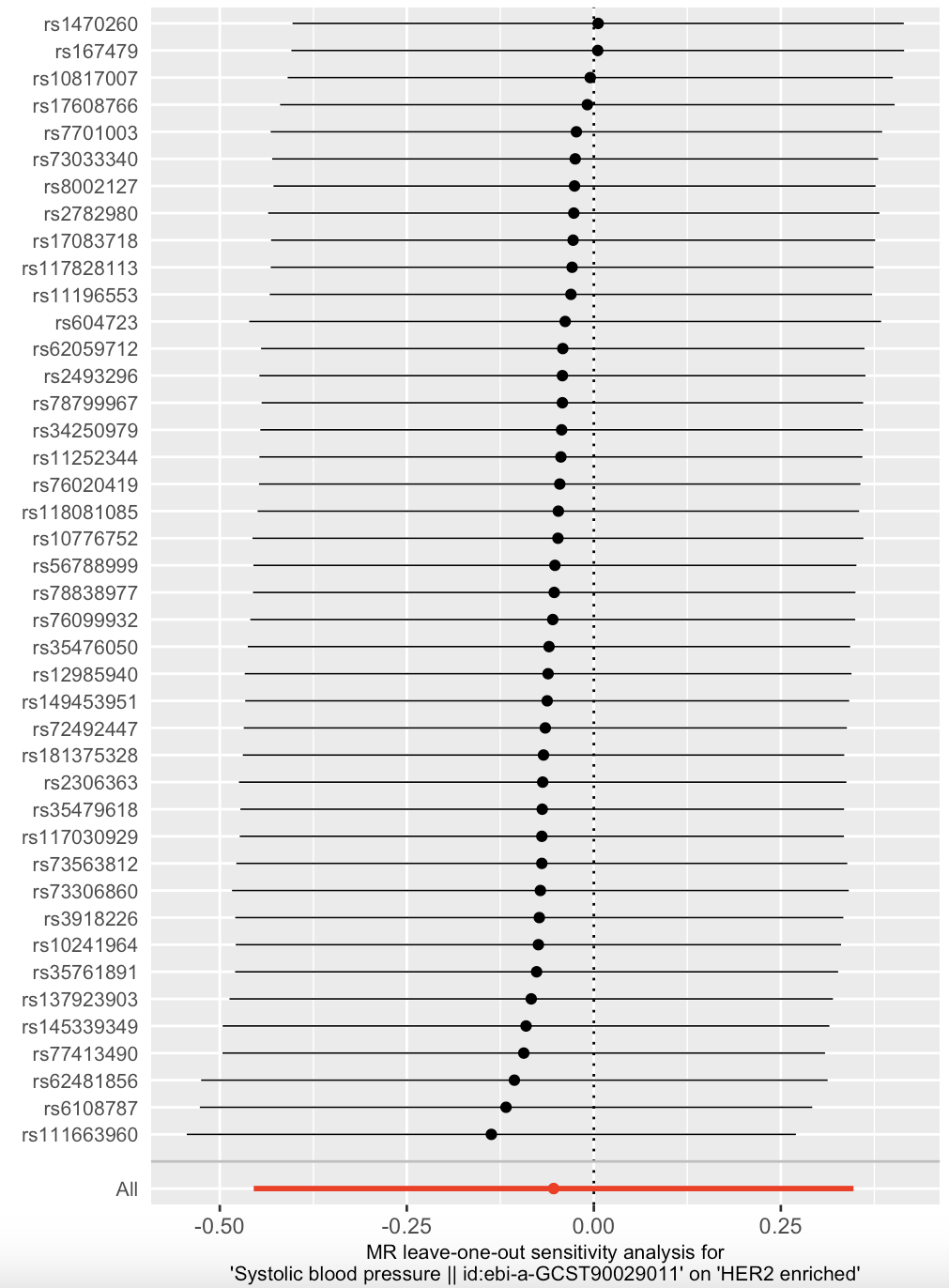

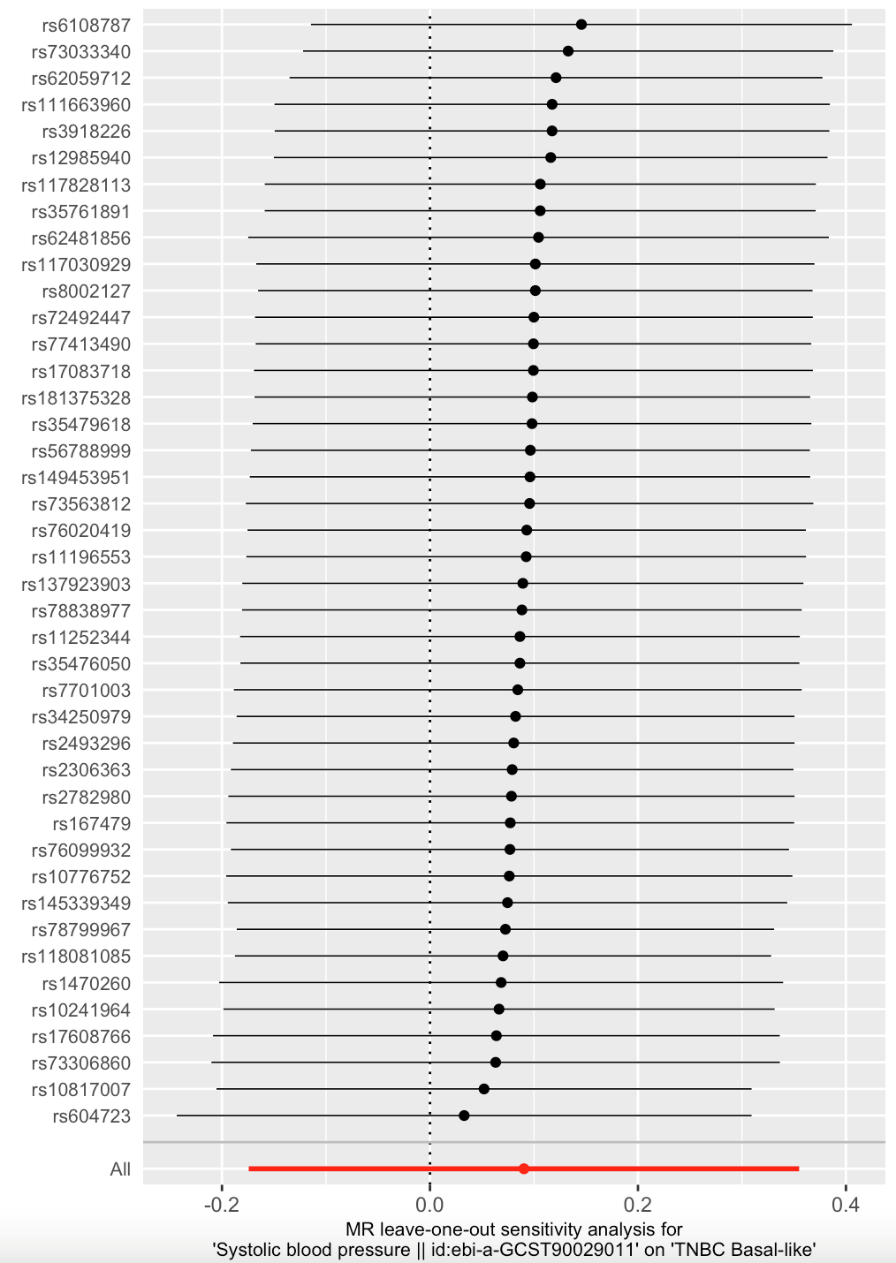
TNBC (restricted 54 SNPs) b. HER2 (restricted 54 SNPs)

c. Luminal-A (restricted 54 SNPs) d. Luminal-B (restricted 54 SNPs)


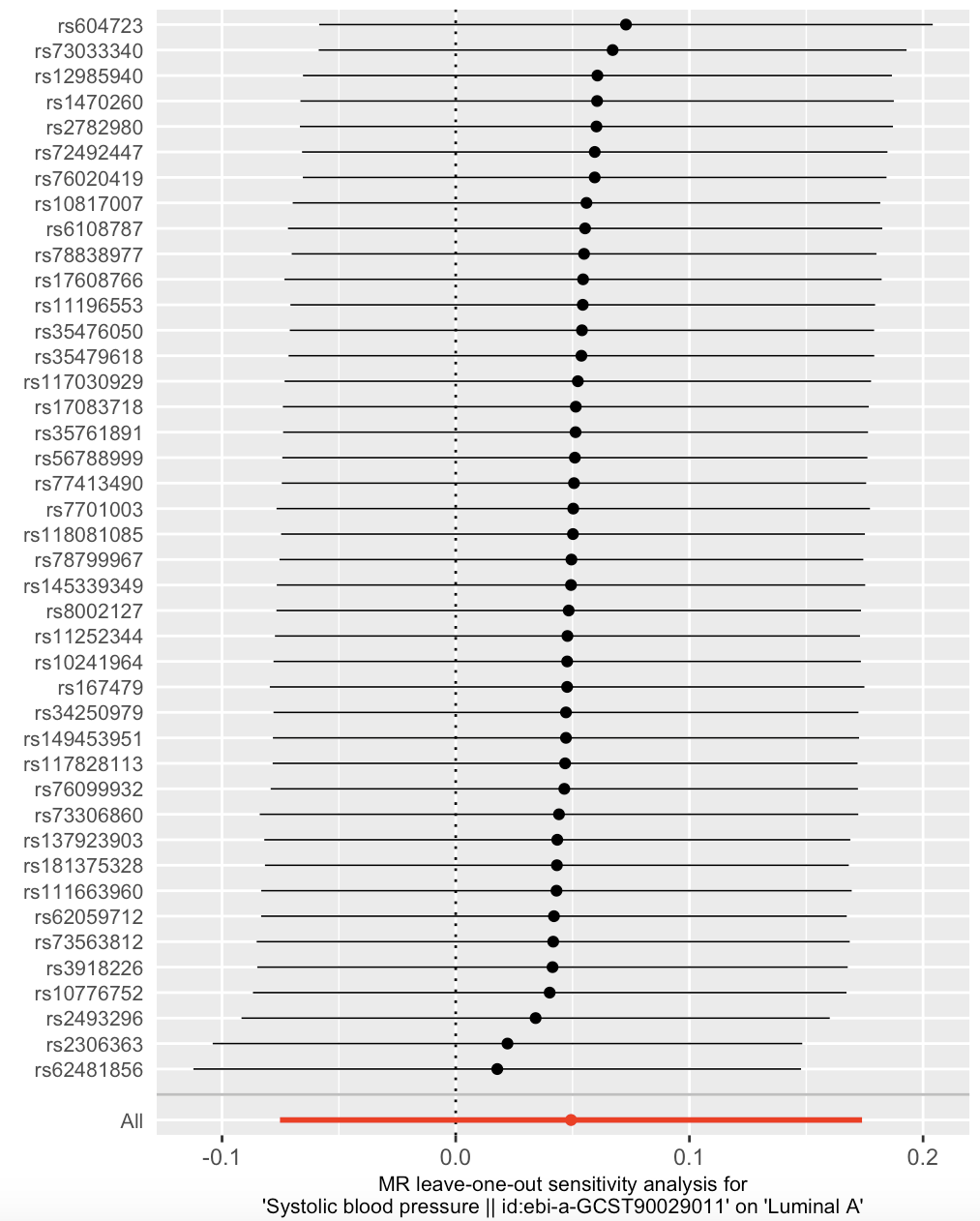

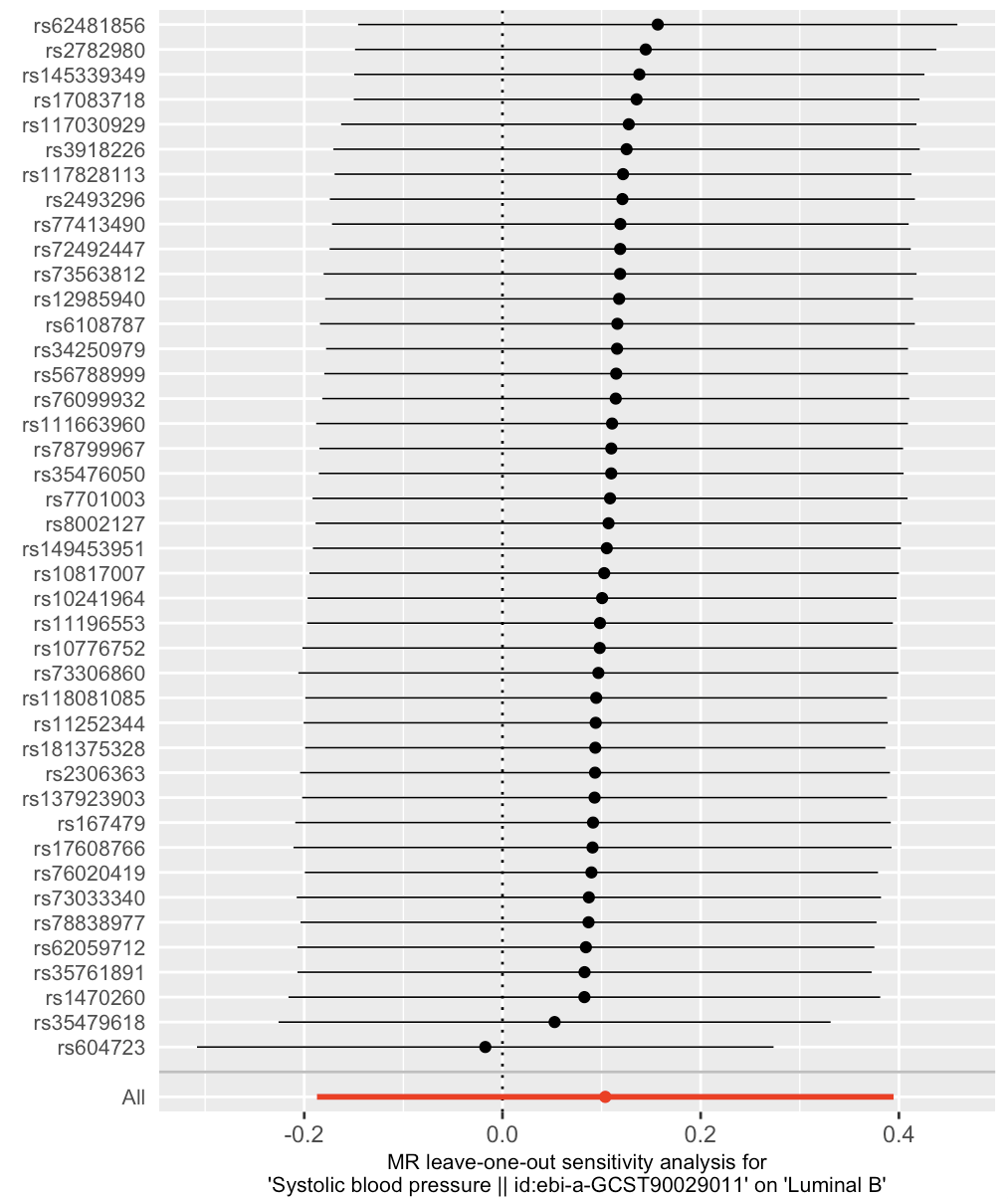


e. Luminal-B HER2

**
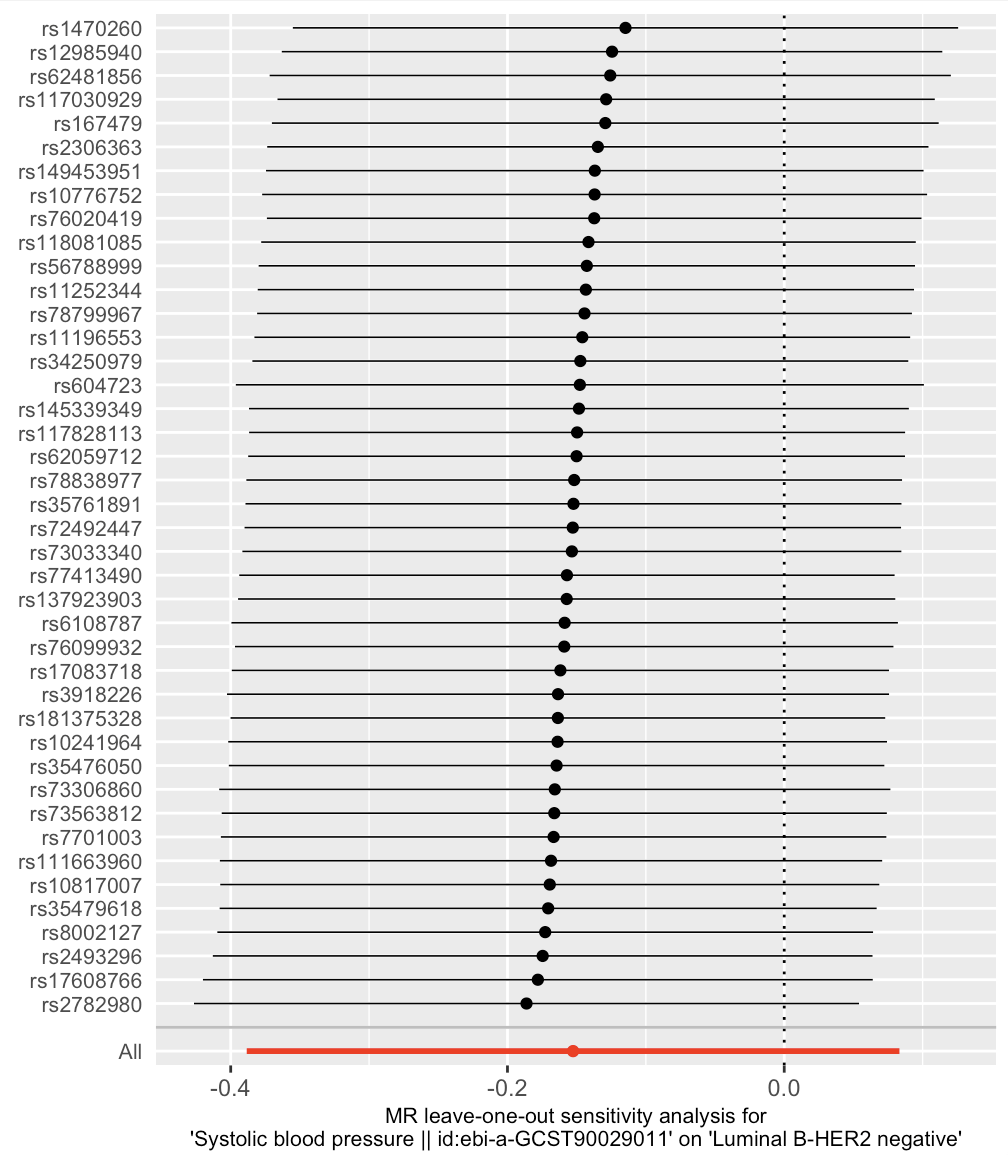
**

**Supplementary figure 3. funnel plot of SBP SNPs and breast cancer**

1. Overall breast cancer


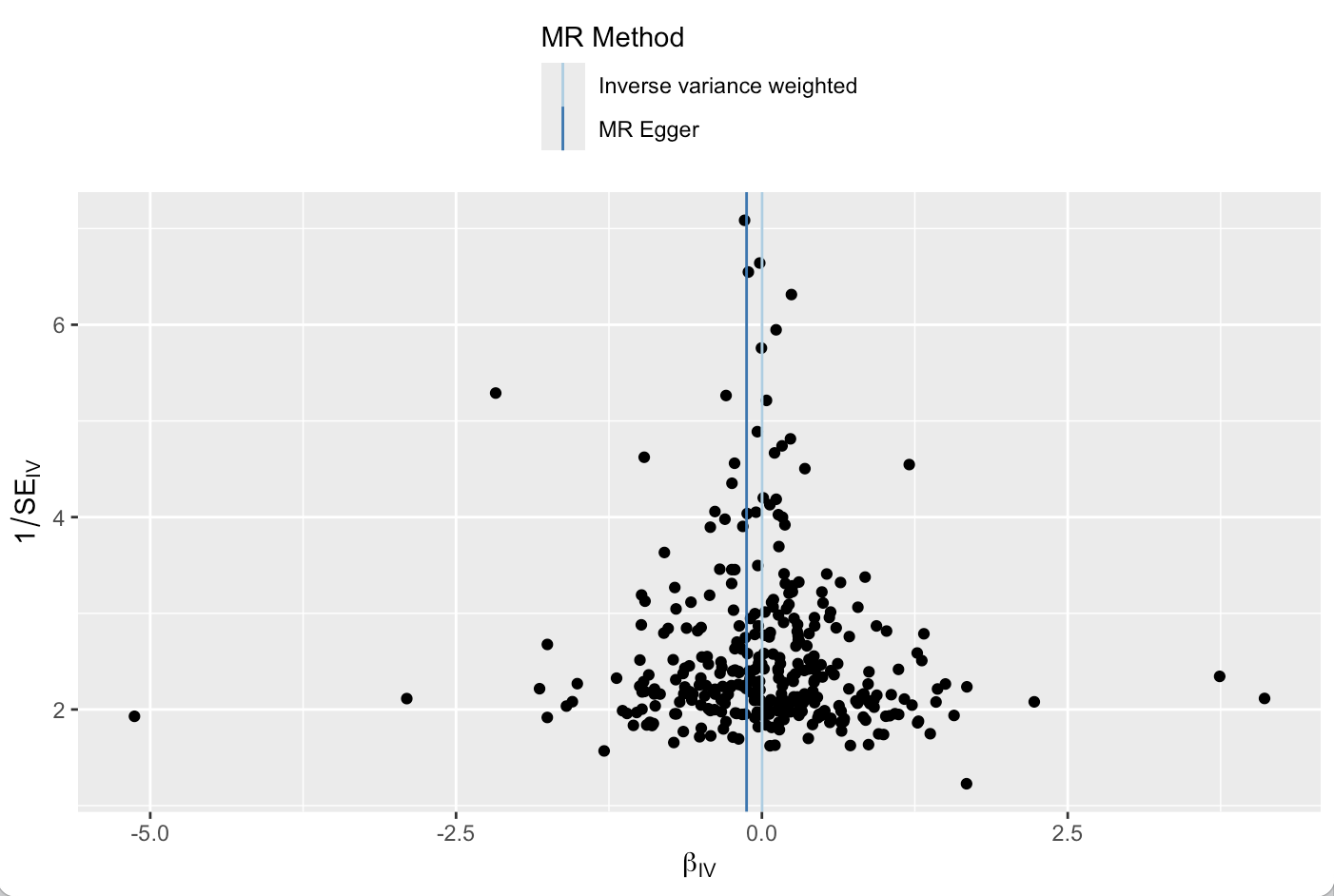


1. **
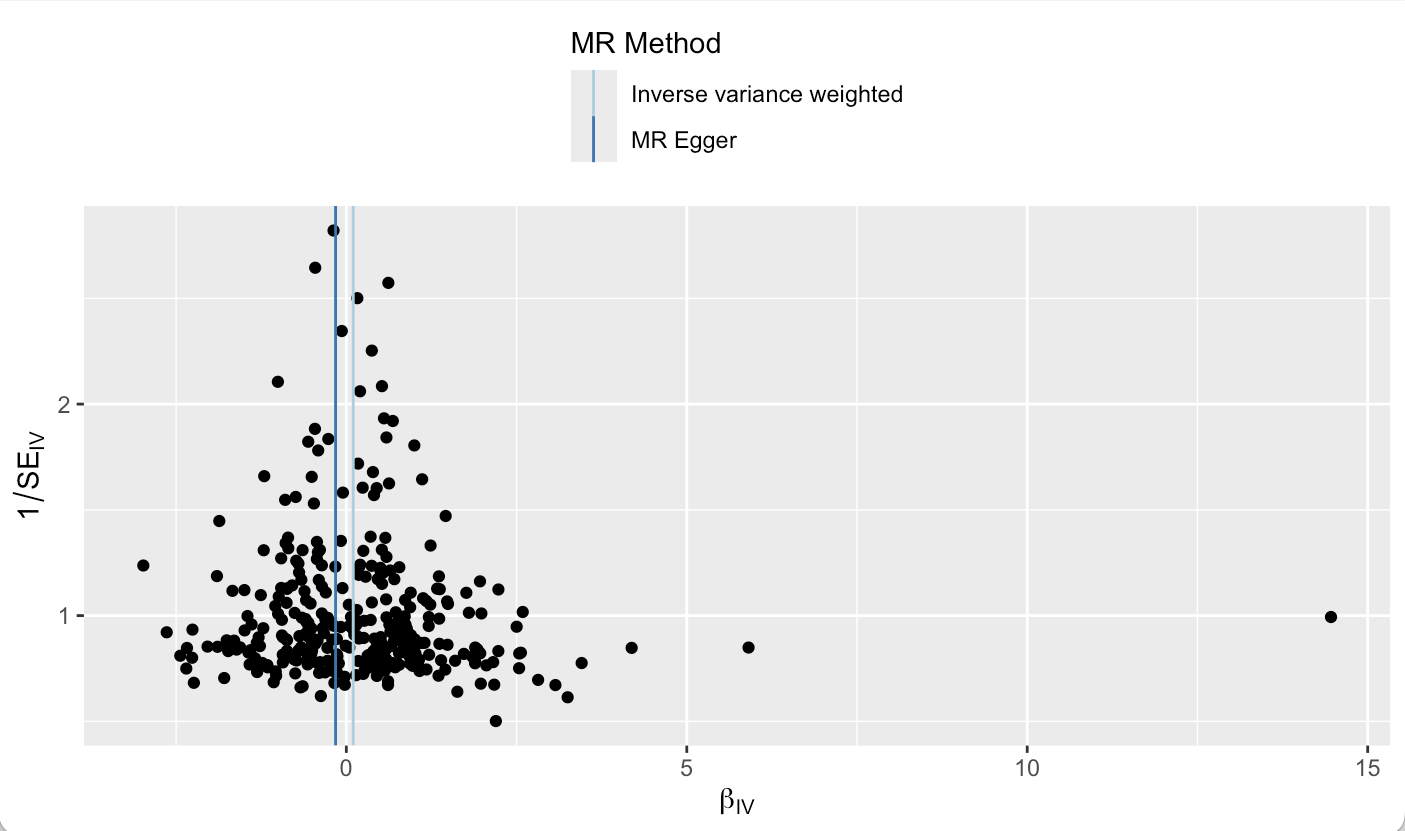
**TNBC
2.
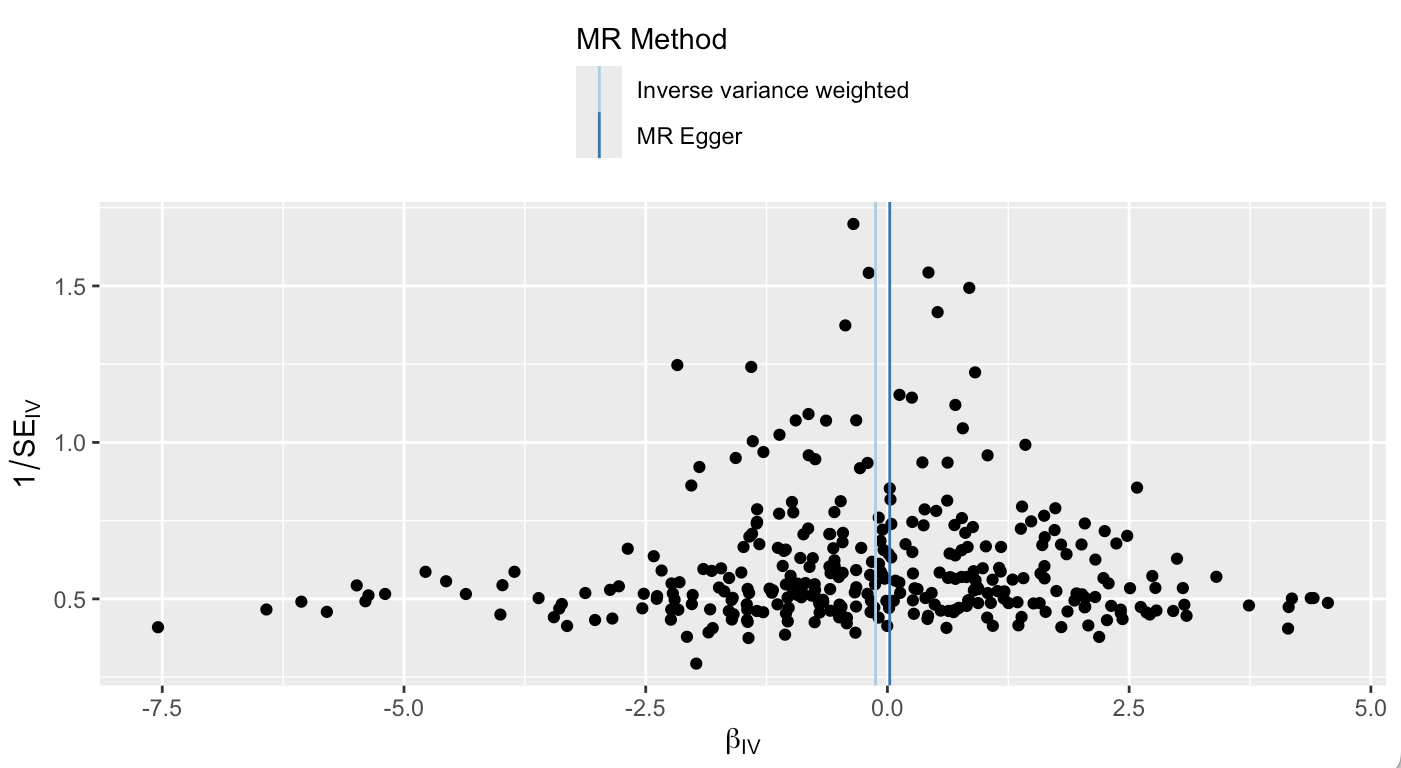
HER2
3. Luminal-A


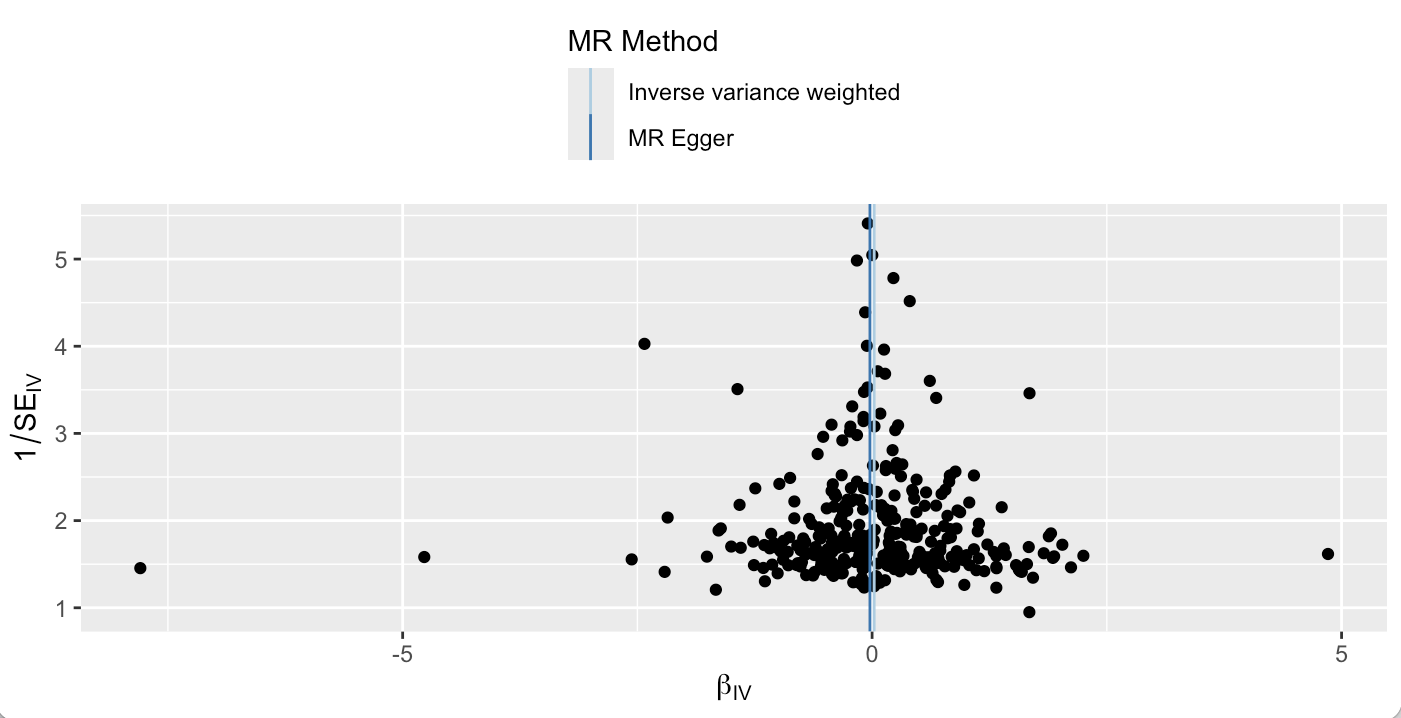


1.
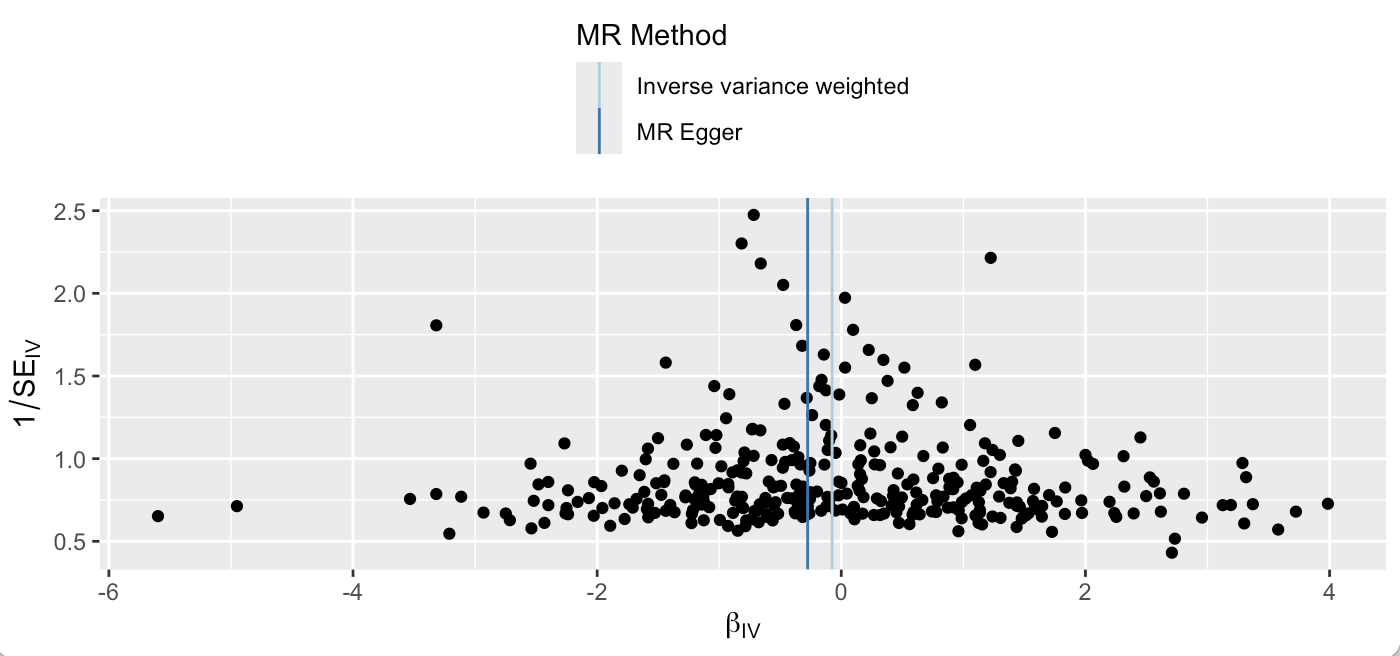
Luminal-B
2. Luminal-B HER2 negative


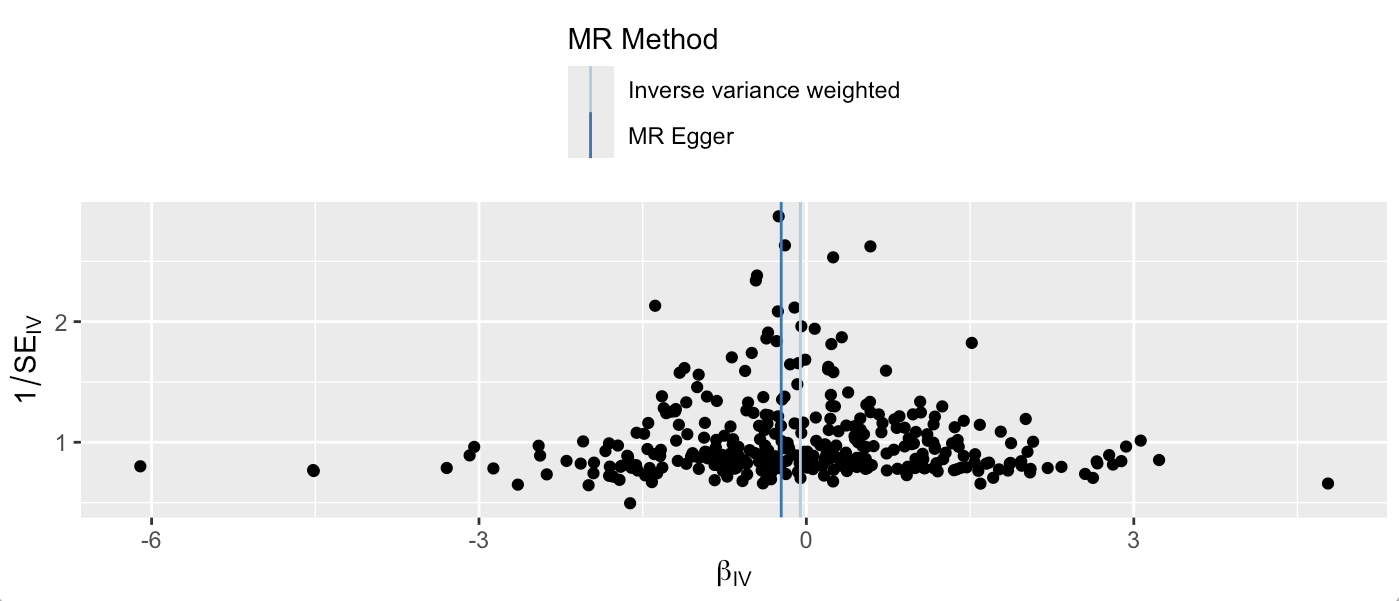

Supplement: Supplementary file 2 — Supplementary Material 2. [file 12885_2025_15513_MOESM2_ESM.docx]
